# Moral echoes: do morality and humanness affect emotional and aesthetic responses to music?

**DOI:** 10.1098/rsos.252167

**Published:** 2026-10-21

**Authors:** Abbigail M. Fleckenstein, Mariola Paruzel-Czachura, Katarzyna Pypno-Blajda, Henna-Riikka Peltola, Jonna K. Vuoskoski

**Affiliations:** ^1^ Department of Psychology, University of Oslo, RITMO Centre for Interdisciplinary Studies in Rhythm, Time and Motion, Oslo, Norway; ^2^ Department of Musicology & Psychology, University of Oslo, RITMO Centre for Interdisciplinary Studies in Rhythm, Time and Motion, Oslo, Norway; ^3^ Penn Center for Neuroaesthetics, University of Pennsylvania, Philadelphia, USA; ^4^ Institute of Psychology, University of Silesia in Katowice, Katowice, Silesian Voivodeship, Poland; ^5^ Department of Psychology, University College of Professional Education, Wrocław, Poland; ^6^ Department of Music, Art and Culture Studies, Finnish Centre of Excellence in Music, Mind, Body and Brain, University of Jyväskylä, Jyväskylä, Finland

**Keywords:** music cognition, moral information, AI-generated music, emotions, aesthetic responses

## Abstract

When listening to novel music, extra-musical information about the musician may influence a listener's evaluations of the music. Here, we studied the extent to which information about the musicians’ Humanness, Morality and Sex, conveyed by their vocal timbre and tessitura, affected listeners’ emotional and aesthetic responses to their music. This online, pre-registered experiment followed a 2 (Humanness: Human vs. AI model) × 2 (Vocalist Sex: Male vs. Female) × 3 (Morality: Moral, Immoral, Neutral) mixed factorial design. Participants (*n* = 400) were randomly assigned to one of four groups, differing in Humanness and Vocalist Sex. Participants read three descriptions depicting a Moral, Immoral and Neutral behaviour and listened to three unfamiliar songs pseudorandomly paired with a description. Emotional and aesthetic responses to each song were measured using validated measures (GEMS-9 scale) and additional items assessing feelings of being Moved, Connected, perceived Beauty, Liking and perceived Creativity. As predicted, linear mixed-effects models revealed significant main effects of Morality for all dependent variables, with Immoral descriptions consistently yielding more negative ratings. Contrary to predictions, we observed no differences in responses to songs based on Humanness, no significant interaction between Morality and Humanness and no effect of Vocalist Sex on responses.

## 1. Introduction

Often, when listening to a new song from an unknown artist, we make snap judgements about the song. These judgements can be informed by several different musical aspects—the genre of the song, the sound of the vocalist(s), the instrumentation or the lyrics of the song—but what kinds of extra-musical aspects about the musicians themselves contribute to and shape these judgements? And can these extra-musical aspects also affect the emotional responses evoked by the music?

Modern consumers have become increasingly more cautious about the products they buy and the media they consume [1,2]. Movements such as #MeToo have shed light on transgressions committed by politicians and celebrities alike, fuelling the phenomenon known as cancel culture [1]. The act of ‘cancelling’ refers to the withdrawal of any kind of material or immaterial support for those who have said or done something that can be considered as (morally) unacceptable or highly problematic, generally from a social justice perspective [3]. Cancel culture exists and spreads particularly on social media platforms, but it has also penetrated some offline contexts, such as universities or museums, in forms of speaker cancellations or public demands regarding ‘cancelling’ artworks, monuments or buildings associated with racism, colonialism or sexism [4]. This phenomenon has also affected the music industry [5], with popular musicians’ actions (both past and present) being called into question and scrutinized by audiences and legal personnel. For instance, in 2021, popular singer-songwriter Billie Eilish was called out for using a racist slur in a video filmed when she was a teenager. This was followed by her fans publicly destroying merchandise and posting on social media for her to be boycotted. Ultimately Eilish publicly apologized for her past behaviour via her official Instagram account [6]. The existence of this phenomenon suggests that, at least to some extent, people care about the moral values of artists and prefer to consume products, media and art created by individuals with similar moral values or, at the very least, those who have not committed moral transgressions.

### 1.1. Foundations of morality

Morality is a broad and multifaceted psychological construct that can be conceptualized in multiple ways depending on the theoretical perspective adopted. For the present study, we adopt the moral foundations theory, one of the most influential frameworks in moral psychology (e.g. [7,8]), which proposes that moral judgements are organized around several distinct but related foundations, including care, fairness (equality and proportionality) [9], loyalty, authority and purity [10]. Of these foundations, some researchers claim that the care foundation plays a particularly central role in moral cognition and consider it the most important moral foundation [11,12], whereas others suggest that fairness is primary for functioning in society [13]. Both are considered individualizing moral foundations, meaning they prioritize an individual's rights and well-being [8] and are closely linked to empathy [14] and compassion [15,16]. Accordingly, we focused specifically on these foundations.

### 1.2. The role of perceived morality in emotional responses

Outside the domain of music, information about the morality of a person has been shown to modulate observers’ empathic responses and felt emotions. Neural, physiological and self-report evidence has revealed that the morality of a person experiencing pain or misfortune affects the intensity of observers’ empathic responses, with immoral targets evoking less empathy than neutral or moral targets [17–19].

Morality is also central to specific types of emotional responses that are often experienced in aesthetic (and non-aesthetic) contexts, such as being moved. Multiple theoretical frameworks highlight moral and altruistic acts as powerful elicitors of being moved [20–22], and empirical work has demonstrated that appraisals of morality significantly predict experiences of being moved, both for observed videos and recalled personal events [23,24]. More broadly, recent theoretical frameworks have conceptualized being moved as a social-relational emotion with social bonding functions—also in the context of the arts [20,25]. Within the *kama muta* framework [20], moral and altruistic acts are argued to evoke the feeling of being moved because they are appraised as instances of communal sharing: the creation, deepening or extension of social bonds. This account is in line with the finding that being moved is typically experienced in situations characterized by increased interpersonal closeness, connectedness and/or moral virtues, such as reunions, critical life events (births and weddings) and witnessing acts of altruism or self-sacrifice [20,25]. Although morality's role in music-induced emotions has yet to be investigated, feelings of being moved elicited by music have been associated with an increased sense of connectedness and social bonding [26,27], similar to feelings of being moved experienced in other contexts [24,25].

Research on the relationship between an artist's morality and audience members’ emotional and aesthetic responses to their work has so far been limited to the domain of visual art, with only the effect of painters’ morality having been evaluated. This research suggests that ratings of liking, quality and beauty for artworks are significantly affected when viewers are given moral or immoral descriptions compared to neutral or ‘factual’ information about the painters, with immoral descriptions leading to lower ratings across these criteria [26–30]. Another recent study builds on these findings, by observing that a painter's moral reputation influenced not only how beautiful participants judged a painting to be but also how viewers felt while engaging with the paintings (spanning positive affect, epistemic transformation, immersion and negative affect) [31]. Specifically, paintings linked to a moral artist increased both the perceived beauty of the painting and the viewer's positive affective response, while paintings linked to an immoral artist intensified negative affective responses [31].

Despite this previous research linking emotional and aesthetic responses and morality with visual artworks, it is not yet known how moral information might modulate aesthetic and emotional responses to music. The question of how the morality of musicians affects listeners’ evaluations of their music remains underexplored. Furthermore, previous work on the effect of moral information on aesthetic responses to visual art has employed rather overt manipulations, presenting large numbers (20–60) of stimuli [30,28] paired with descriptions portraying extreme (im)moral behaviour (e.g. supporting Nazi policies or committing child abuse) [29]. Such high-intensity, explicit manipulations may not accurately capture the more nuanced ways moral considerations operate in real-world art and music consumption.

Importantly, moral judgements about others are shaped not only by a person's actions or behaviours but also by certain demographic criteria. Research shows that a person's sex or gender is a highly salient identity cue that influences person perception and moral evaluation [32]. Women are often evaluated more favourably than men on broad social and affective dimensions such as warmth [33], and they are also more strongly associated with specific moral traits such as trustworthiness [34]. At the same time, these positive moral expectations may create a higher ethical standard for women. For example, some studies suggest that ethical conduct is more strongly prescribed for women than for men, and that women may be punished more severely following ethical or integrity violations [35,36]. Applied to music listening then, this previous research might suggest that moral or immoral behaviour may be judged by listeners differently depending on the sex of the musician. Specifically, female musicians may be held to a higher moral standard, meaning that if immoral behaviour is attributed to them, this could elicit stronger negative responses from listeners compared to the same immoral behaviour attributed to male musicians.

### 1.3. The role of perceived human agency in music

Music achieves many of its emotional effects by activating the same processes and capacities as those involved in social interaction, including empathy and social cognition [37–39]. The main theoretical frameworks of music-induced emotion outline empathy and emotional contagion as some of the key mechanisms through which music induces emotional responses in listeners [37,40,41]. Furthermore, many of people's most cherished musical experiences, whether at live concerts or alone at home listening to recorded music, involve a deep feeling of human connectedness and understanding [26,27]. Musicians and composers represent tangible human agents, whose intentions and felt emotions can be understood and empathized with through embodied and imaginative processes [37–39]. Music may also convey a more emergent sense of agency through learned associations between musical sounds and human motor actions, the hierarchical organization of sounds and the elicitation of motor resonance [42]. But does our experience of—and engagement with—music change when we know its creator is not a human agent?

The landscape of music composed using artificial intelligence (AI) is rapidly developing. With this, there has also been an increase in empirical research evaluating individuals’ reactions to music purportedly created using AI. Some of this research suggests a bias against AI-generated music, whether based on labelling alone [43] or actual AI authorship [44], with human-labelled or human-created music rated as more liked, more engaging and exhibiting higher quality [43,44]. Previous work has also shown that beliefs about a human (versus computer) composer can modulate the activation of brain areas related to social cognition during music listening, highlighting the involvement of processes such as mental state attribution and the detection of intentionality in musical engagement [45].

Other studies on the same topic have obtained more mixed results. Friedman & Taylor [46] found that listeners’ liking and quality ratings for ‘happy’ music were higher if the music they listened to was labelled ‘human composed’ compared to ‘computer composed’, but no significant differences were observed regarding their ratings of subjective arousal, valence, enjoyment and interest. Other research indicates that differences between listeners’ liking and quality ratings of music labelled AI- or human-created may be contingent upon the musical genre of the songs. For example, ratings for classical music were significantly lower when listeners were told that the music was created by AI, but ratings for electronic music did not significantly differ based on the listeners being told the music was created by AI versus humans [47].

In addition to these inconsistent findings from previous research on responses to AI music, several knowledge gaps exist. Namely, listeners’ felt emotions and aesthetic responses are seldom measured in these studies and are limited to ‘arousal’ and ‘valence’ dimensions, and the songs used in existing studies are limited to instrumental music.

### 1.4. The present study

We aimed to evaluate the extent to which a musician's morality and humanness influence listeners’ felt emotional and aesthetic responses to their songs. The experimental design, research questions, hypotheses and analysis plan for this experiment were pre-registered on OSF on 24 April 2025 ( https://doi.org/10.17605/OSF.IO/W7346). In this study, Humanness refers to the literal quality of being human, as opposed to a generative AI model, and Morality refers to the quality of behaving in moral (i.e. good) or immoral (i.e. bad) ways. This study aimed to systematically manipulate aspects of Humanness and Morality by providing a short description of either a Human musician or AI model depicting different actions (Moral, Immoral or Neutral), meaning that Humanness was manipulated by labelling used in the written descriptions only. With the intention of extending previous work on the effect of moral information on the aesthetic evaluation of visual art, we attempted to use more subtle manipulations [cf. 29] using active deception and descriptions that included other biographical details in addition to information about Moral or Immoral behaviour. The influence of these manipulations was investigated by analysing listeners’ emotional and aesthetic responses provided to songs reported to be created and performed by either a Human or AI model. Accordingly, the three main research questions and corresponding hypotheses for this study were as follows:

RQ1: Do listeners’ emotional and aesthetic responses to music differ when the music is believed to be composed by a generative AI model compared to a human musician?

H1: Listeners will provide higher emotional and aesthetic response ratings to music believed to be composed by a human musician compared to a generative AI model.

RQ2: Does the moral depiction of a human musician/generative AI model impact listeners’ emotional and aesthetic responses to their music?

H2: Relative to the neutral depiction, moral depictions will lead to higher emotional and aesthetic response ratings, while immoral depictions will lead to lower ratings.

RQ3: Does the Humanness of the song's creator (human musician/generative AI model) moderate the effect of moral depictions on listeners’ emotional and aesthetic response ratings?

H3: The Humanness of the song's creator will moderate the effect of moral depictions on listeners’ emotional and aesthetic response ratings, and the effect will be stronger when the song's creator is depicted as a human musician compared to a generative AI model.

While the main focus of this study was to evaluate the influence of Morality and Humanness on emotional and aesthetic responses, the songs chosen for this experiment incorporated vocalists, thereby introducing a third aspect—the sex of the vocalist as conveyed by their vocal timbre and tessitura. The choice to use songs that incorporated a vocalist was motivated by a desire to (i) extend previous research evaluating listener responses to music believed to be AI-generated, which has mostly implemented instrumental music [see 43,44,46,47]; (ii) reinforce the believability of the descriptions of human musicians; and (iii) anthropomorphize the songs believed to be AI-generated. To account for the potential influence the vocalist's sex may have on listeners’ responses, female and male versions of each song were included in the experiment, and an ancillary fourth research question and a non-directional hypothesis were added:

RQ4: Does the sex of the vocalist moderate the effect of moral depictions on listeners’ emotional and aesthetic response ratings?

H4: The sex of the vocalist will interact with moral depictions of human musicians and have an effect on listeners’ emotional and aesthetic response ratings.

This fourth research question was only applied to the depictions of Human musicians, as it was unclear to what extent Vocalist Sex would interact with Moral depictions of generative AI models. The choice to make H4 non-directional was made as a result of women being both associated with higher moral traits [34] and also punished more severely for moral transgressions [35,36].

## 2. Methods

### 2.1. Design

The study was implemented as an online experiment that followed a 2 (Humanness: Human vs. AI) × 2 (Vocalist Sex: Male vs. Female) × 3 (Morality: Moral, Immoral, Neutral) mixed factorial design. As illustrated in figure 1, the first and second factors, Humanness and Vocalist Sex, were implemented as between-subjects factors, and both had two levels: Human musician versus generative AI model and Male versus Female. The third factor, Morality, was implemented as a within-subjects factor with three levels: Moral, Immoral and Neutral.

**Figure 1.**
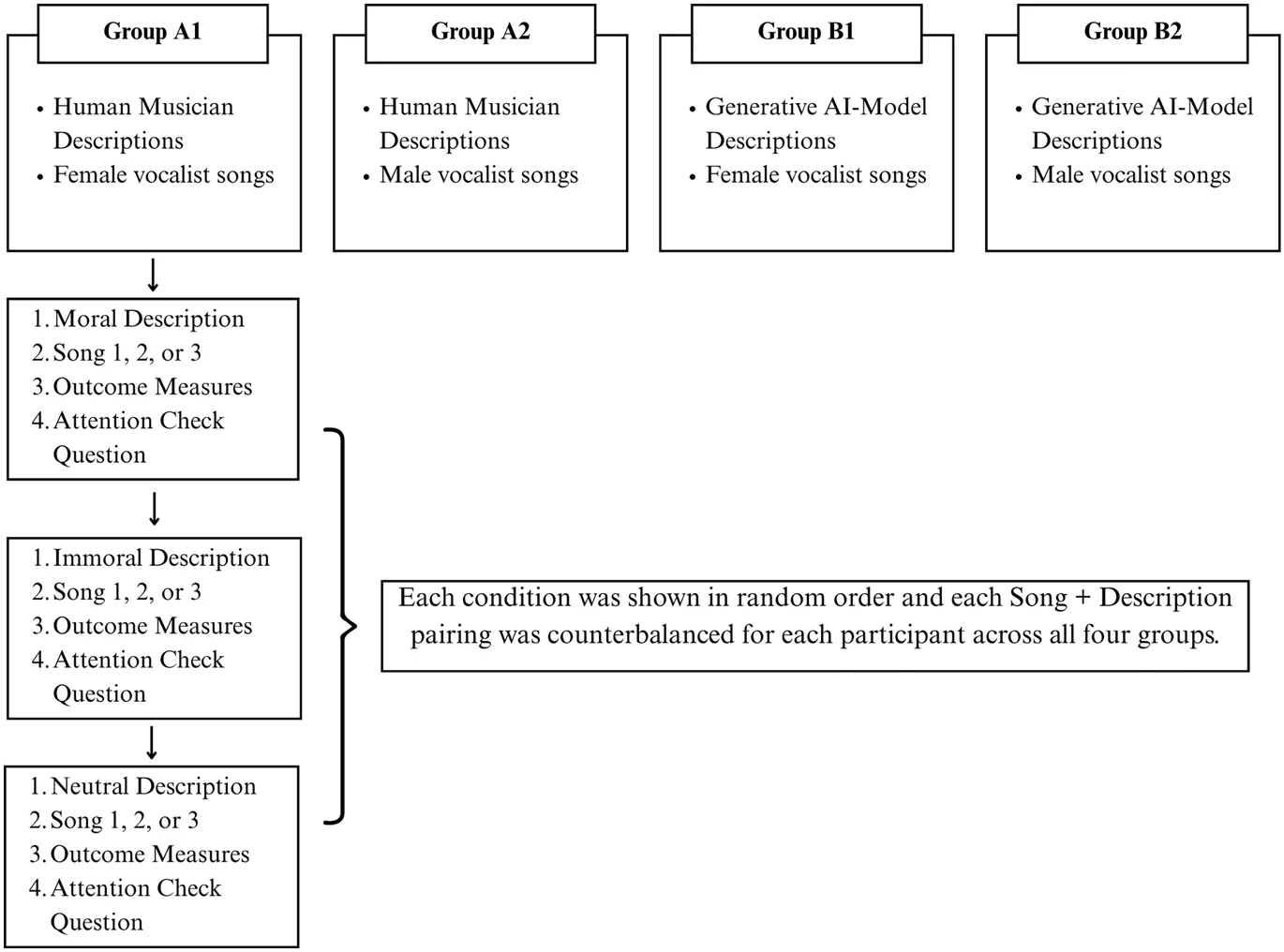
Experimental design. The presentation order of the three morality conditions was randomized for each participant, and the song + description pairings were pseudorandomized and counterbalanced for participants across the four groups such that each song appeared equally often with each morality description.

Each of the four participant groups partook in the Moral, Immoral and Neutral conditions, presented in random order, with the song-description pairings pseudorandomized and counterbalanced across participants to avoid sequence effects. The decision to treat Humanness and Vocalist Sex as between-subjects factors and Morality as a within-subjects factor was made to strengthen the believability of the descriptions participants read, minimize confounding Vocalist Sex with Morality and limit the number of stimuli presented to each participant.

### 2.2. Sample size estimation

The sample size for this study (*n* = 400) was determined by *a priori* power and sensitivity analyses conducted using G*Power 3.1 to estimate the required sample size for testing the aforementioned hypotheses. We used a mixed ANOVA design to approximate the required sample size for a linear mixed-effects model analysis [for a similar approach, see 48]. These analyses indicated that a minimum of 176 participants per factor level (i.e. 352 participants in total for a between-subjects factor with two levels) were required in order to have 80% power to detect a small-to-medium effect of *f* = 0.15 at a 0.05 alpha error rate to test H1 (Humanness). The predicted effect size was based on a previous study by Shank and colleagues [study 3,47]. In order to accommodate potential missing data and exclusions, we aimed to recruit a minimum of 400 participants. Subsequent sensitivity analyses also indicated that a sample of 400 participants would provide 80% power to detect a very small effect of *f* = 0.06 at a 0.05 alpha error rate in a within-subjects factor with three levels (H2; Morality), as well as in a two-way within–between interaction (H3; Morality × Humanness). Additional analyses also indicated that having a sample of 200 participants (i.e. those participants who are assigned to the Human Musician conditions) to test the two-way within–between interaction between Morality and Vocalist Sex (H4) will provide 80% power to detect a small effect of *f* = 0.09 at a 0.05 alpha error rate. An overview of the exact power analysis protocols followed for this study can be found on OSF (https://osf.io/y85kx/files/wr4b6?view_only=c940399c547244e5bc17f110adf8fa07).

### 2.3. Participants

After obtaining ethical approval from the Internal Ethics Committee of the Department of Psychology at the University of Oslo, a total of 400 native English-speaking participants living in the United States or the United Kingdom were recruited through Prolific, an online paid participant recruitment platform [49,50]. The total sample of participants (*n* = 400) was between the ages of 19 and 83 (*M* = 42.12, *s.d.* = 13). Using Prolific's quota sampling, a 50/50 balance between male and female participants was set for each of the four participant groups. As a result, 198 participants in the experiment identified as male and 201 as female.^1^ An overview of participants’ age and sex is shown in table 1. Each participant received £3 for completing the questionnaire.

**Table 1.** Participant demographics.

participant group	*N*	Age		Sex		
		mean	s.d.	Male	Female	prefer not to disclose
**Group A1**	100	42.63	12.23	47	53	0
**Group A2**	100	44.56	13.69	50	50	0
**Group B1**	100	39.33	12.57	51	49	0
**Group B2**	100	41.99	13.14	50	49	1

*Note:* s.d. = standard deviation. Participants were given the option to not disclose their sex in the present experiment. However, Prolific's quota sampling is based on demographic information provided by users directly to the platform.

### 2.4. Procedure

After providing informed consent, participants were shown a fake title for the study, ‘How Music Affects Memory’, and were given a false cover story explaining that the study aimed to ‘learn more about how music listening interacts with short term memory’. Following this, participants completed the three conditions in random order. In each condition, participants first read a short description depicting moral, immoral or neutral actions of either a human musician or a generative AI model. They then listened to a song with Norwegian lyrics, featuring either a male or female vocalist, presented as having been created and performed by the described musician. After each song, participants were asked to provide ratings of their felt emotional and aesthetic responses to the song and answered an attention check question about the description.

Following the presentation of the three conditions, participants’ age, sex assigned at birth and trait empathy [51] were recorded. Lastly, before the debrief statement was provided, participants were asked an open-ended deception check question: ‘On the following page you will be informed of the full aims and intent of this study. Before this, do you have any questions or comments about what this study is about?’ The true aims of the study were then disclosed, including the use of active deception. Before submitting their survey responses, participants were given the opportunity to withdraw their consent; however, no participants chose to do so.

## 3. Material

### 3.1. Descriptions

Morality was manipulated by altering the actions of the human musicians or generative AI models depicted in the descriptions. The descriptions used in the present study were chosen on the basis of a pilot study (*n* = 52) which included 18 descriptions created by the research team to either uphold or violate principles belonging to the Care and Fairness foundations of the Moral Foundations Theory [7]. The most believable and effective Moral, Immoral and Neutral descriptions for both Human musicians and generative AI models were selected to be included in the main study, with one exception: the second most effective Immoral AI description was selected to maintain consistency with the other descriptions, which belonged to the Care foundation. All six descriptions included in the main study can be found on OSF (https://osf.io/tkgu6?view_only=c940399c547244e5bc17f110adf8fa07). Three examples of these are mentioned in the following sections.

#### 3.1.1. Immoral human description

This musician, a 38-year-old folk-pop artist from Oslo, has been a prominent figure in the Norwegian music scene for over a decade. Their annual summer tour, typically covering 15 cities across the country, has been a beloved tradition. However, it has recently been uncovered that the musician has been exploiting vulnerable individuals who sought their mentorship and guidance in the industry. Aspiring artists working as stagehands for this musician have come forward with allegations of emotional manipulation, verbal abuse and even instances of physical intimidation spanning several years. The revelation came after several victims shared their experiences online, prompting an investigation by Norwegian authorities.

#### 3.1.2. Moral AI description

This AI model, developed by a team of researchers at a university in Oslo over the past three years, analyses the emotional responses of listeners and, using research-based practices endorsed by Norwegian music therapists, creates songs that aim to provide comfort and solace to those experiencing sadness or grief. The AI has been used in over 50 mental health clinics across Norway. Its user base primarily consists of mental health professionals and individuals seeking personalized emotional support through music.

#### 3.1.3. Neutral AI description

Originally developed in Oslo, this AI has been used by people both nationally and internationally over the last three years. The AI specializes in generating pop, folk and lo-fi music. It has gained popularity among independent filmmakers, content creators and app developers. The AI has been used to create over 10 000 unique compositions, which have been accessed by users in over 20 countries. Its primary user base is located in Scandinavia but has expanded to other European countries.

### 3.2. Musical stimuli

#### 3.2.1. Selection

The three songs used in this experiment were chosen based on results from a second pilot study (*n* = 50) which included eight songs chosen from artlist.io, a subscription-based online platform with royalty-free music. These eight songs were included in the second pilot study as they contained a vocalist, belonged loosely to the singer-songwriter pop genre, were shorter than 3 min 30 s and displayed good singability, aesthetic appeal and potential to evoke feelings of being moved.^2^ These eight songs were originally written and recorded by human musicians with English lyrics. To create male and female versions of these songs, the original vocal tracks were replaced with different male and female voice clones that were applied using an AI-powered voice conversion platform, audimee.com.^3^ The male and female voice clones were chosen for each song based on the fit between vocal and instrumental timbre, evaluated by the first author who is a trained choral instructor. Pilot study participants were presented with either the male or female version of each song and were then asked to rate 12 items measuring their felt emotions and aesthetic responses (GEMS-9 Scale, feeling Moved, Beauty, Liking and Creativity) to each song.

Effect sizes (Cohen's *d*) were calculated for the 12 items rated for the male and female versions of the eight songs in the pilot study. The averages of the absolute Cohen's *d* values across all 12 items rated were then determined for the male and female versions of the eight songs, and the three songs which had the smallest differences between the male and female versions, as measured by effect size, were then selected for the main study. This decision was made to limit extraneous variables, such as preference for a specific vocal timbre, that may have led to higher or lower ratings for the female version of the song but not the male version or vice versa. The three songs chosen were ‘Broken Leg’ originally by Straight White Teeth (Song 1), ‘Castaway’ by Lightboys (Song 2) and ‘Endless Summer’ by Team Callahan (Song 3), all of which had a small effect size, *d* < 0.18, indicating small differences between ratings for the male and female versions of the songs. Two potential limitations of this pilot study should be noted. First, participants were not asked to rate the degree to which they believed the songs to be performed by a human or AI. Second, the songs in the pilot study had English lyrics, unlike the main study, meaning that the lyrical content may have influenced participants’ ratings in the pilot study. However, this influence would have affected both the male and female versions of the songs.

#### 3.2.2. Editing

The vocal tracks for the three songs chosen from the pilot study were then edited. The song lyrics for each of the three songs were transcribed and translated from English into Norwegian, and new Norwegian vocal tracks, performed by native Norwegian-speaking singers, were recorded. The choice to translate the songs was to prevent any potential confounds resulting from understanding the songs’ lyrics that may have interacted with the Morality manipulations (i.e. the descriptions). The Norwegian language was chosen as it was considered to be unlikely that American and UK-based participants would be able to understand the Norwegian song lyrics [52]. Once the Norwegian vocal tracks were recorded, the same six voice clones (three male and three female) that were applied to the English vocal tracks in the pilot study were then applied to the Norwegian vocal tracks. This procedure created Norwegian versions of the songs that were closely matched to the English versions used in the pilot study, having the same cloned voices. The final, voice-cloned versions of the three songs used in the main study can be found on OSF (https://osf.io/be6g5?view_only=c940399c547244e5bc17f110adf8fa07).

### 3.3. Online survey

Data collection took place between 7 and 9 May 2025, and consisted of online surveys hosted on Qualtrics. A total of four surveys were created, one for each participant group. The four survey versions can be found on OSF (https://osf.io/y85kx/files/osfstorage?view_only=c940399c547244e5bc17f110adf8fa07). At the start of each survey, participants were asked to verify that they could not speak or understand Norwegian.

Felt emotional and aesthetic responses were measured by 14 items rated using a 100-point visual analogue scale. These items included the GEMS-9 scale [53], measuring music-induced emotions: Wonder, Transcendence, Peacefulness, Tenderness, Nostalgia, Power, Joyful Activation, Sadness, Tension and feelings of being Moved, which was treated as an independent item owing to its relevance to moral appraisal [24]; as well as Beauty, Liking, feeling Connected and perceived Creativity. No definitions were provided for any of the items measured. However, the GEMS-9 scale lists additional terms related to the main rated item (e.g. ‘**Wonder**: wonder, dazzled, allured’). For the non-GEMS items, participants were asked: ‘How beautiful do you think the song was?’, ‘How much did you like the song?’, ‘How connected did you feel to the music?’ and ‘How creative do you think the song was?’.

The attention check questions at the end of each condition served as a quality control measure to verify that the descriptions had been read and to maintain the false cover story about the effects of music listening on short-term memory. Trait empathy was assessed using the seven-item Empathic Concern subscale of the Interpersonal Reactivity Index (IRI), which gauges one's tendency to experience sympathy, compassion and concern in response to others’ experiences and to care about their welfare [51]. Empathic Concern was selected over the entire IRI because this specific subscale has been found to robustly predict the tendency to experience being moved [54]—one of our main dependent variables—and it also predicts sensitivity to moral information [55].

## 4. Analysis

### 4.1. Indices

Indices were created for the three factors of the GEMS-9 scale [53] by averaging the ratings for each item comprising each factor. As such, the ratings for wonder, transcendence, peacefulness, tenderness and nostalgia items were averaged to create a Sublimity Index,^4^ ratings for power and joyful activation items were averaged to create a Vitality index, and the ratings for sadness and tension items were averaged to create an Unease index.

### 4.2. Linear mixed-effects models

Two linear mixed-effects models were fit to each of the eight dependent variables constituting listeners’ emotional and aesthetic response ratings: the three GEMS-9 indices (Sublimity, Vitality and Unease) and the five additional items measuring feelings of being Moved, Beauty, Liking, Connected and Creativity using the lme4 package [56] for R v.4.3.1. Model 1 tested the pre-registered hypotheses 1–3 by examining the effects of Morality and Humanness on listeners’ emotional and aesthetic response ratings with random intercepts included for participants. The pre-registered analysis plan to treat ‘Music_ID’ as a random effect (https://doi.org/10.17605/OSF.IO/W7346) produced singular fit warnings and failed to account for the dependency between the male and female versions of each song. Therefore, we deviated from the preregistration and updated Model 1 (shown below) to include random intercepts for songs nested within vocalist sex, (1|Vocalist_sex/Music_ID), following recommendations for maximal random effects structures [57]. However, this random effects structure also produced singular fits for several dependent variables, indicating overparameterization [56]. Following standard practice [58], we then simplified the random effects structure for each affected dependent variable using an iterative or tiered approach. As a result, the structure of Model 1 was simplified to Model 1a (shown below) for Sublimity, Vitality and Unease. Model 1a was further simplified to Model 1b (shown below) for Creativity, which had persistent singular fit warnings.

**Model 1. Maximum Random Structure: Moved, Beauty, Connected and Liking**
DV ∼ Morality × Humanness + (1|Participant_ID) + (1|Vocalist_sex/Music_ID)
**Model 1a. Unnested Random Structure: Sublimity, Vitality and Unease**
DV ∼ Morality × Humanness + (1|Participant_ID) + (1|Music_ID)
**Model 1b. Simplified Structure: Creativity**
DV ∼ Morality × Humanness + (1|Participant_ID)

Model 2 tested hypothesis 4 and examined whether the sex of the vocalist moderated the effect of morality on listeners' emotional and aesthetic response ratings in the human musician participant groups. Model 2 included random intercepts for participants and the three songs, ‘Music_ID’. Owing to treating ‘Vocalist_Sex’ as a fixed effect in this model, it was not possible to incorporate the same nested random effects structure, (1|Vocalist_Sex/Music_ID), used in Model 1. Model 2 also produced singular fit warnings for two of dependent variables, Liking and Creativity. We therefore simplified the random effects structure for these dependent variables in Model 2a (shown below). Only the data from the human musician participant groups were used in Model 2 as there was no clear rationale for why the depicted sex of the vocalist would interact with moral descriptions of generative AI models. On this, further supplementary analyses conducted on all four participant groups after the planned analysis found no significant two-way interaction between Humanness and Vocalist Sex, nor any significant three-way interaction between Morality, Humanness and Vocalist Sex. Results from these supplementary analyses (supplementary materials, tables S1 and S2) indicated that the effects of the vocalist's sex did not differ between the human and AI participant groups.

**Model 2. Maximum Random Structure: Sublimity, Vitality, Unease, Moved, Beauty and Connected**
DV ∼ Morality × Vocalist_Sex + (1|Participant_ID) + (1|Music_ID)
**Model 2a. Simplified Structure: Liking and Creativity**
DV ∼ Morality × Vocalist_Sex + (1|Participant_ID)

For both Models 1 and 2, Satterthwaite's method was used to estimate degrees of freedom and obtain *p*-values for fixed effects [59]. For each model, marginal and conditional *R*² values were calculated to determine the level of variance explained by both fixed and random effects, respectively [60]. Additionally, Benjamini–Hochberg corrections were applied to each individual model across all dependent variables to control for false discovery rate (FDR) when testing the same model across various dependent variables [61]. This addition of the Benjamini–Hochberg corrections to both Models 1 and 2 also deviates from the pre-registered analysis plan (https://doi.org/10.17605/OSF.IO/W7346).

### 4.3. Post hoc pairwise comparisons

For instances where significant interactions and significant main effects were observed for Models 1 and 2, post hoc pairwise comparisons were conducted between each of the three morality conditions using the emmeans package [62] with Holm–Bonferroni corrections for multiple comparisons.

### 4.4. Exploratory analyses

After conducting the two pre-registered analyses, three additional exploratory analyses were performed. The first exploratory analysis investigated whether participants’ trait empathy moderated the effects of Morality, Humanness and their interaction on participants’ felt emotional and aesthetic responses owing to its established associations with the intensity of music-induced emotions [26,63] and experiences of being moved [54]. The second exploratory analysis investigated whether participants’ age moderated the effects of Morality, Humanness and their interaction on participants’ responses. The third exploratory analysis excluded participants who correctly or closely guessed the true intention of the experiment prior to the disclosure of the debrief statement and then fitted the pre-registered models (Models 1 and 2) to the new, robust dataset.

## 5. Results

### 5.1. Quality control

Prior to conducting the planned analyses, each condition in which participants failed the attention check question was removed from the data. This was done as we could not guarantee that the descriptions were read or effective for these particular conditions. Participants could fail a maximum of two out of the three attention check questions. Participants who failed all three attention check questions were rejected from the experiment during the data collection process, and new participants were automatically recruited via Prolific. A total of 68 of the 1200 conditions were excluded (table 2). These exclusions resulted in a slightly unequal number of conditions per participant group. However, the linear mixed-effects models used in the present analyses were able to account for uneven groupings, and the total number of excluded conditions represents a minimal loss of data (5.7%).

**Table 2.** Excluded conditions owing to failed attention check questions.

participant group	Moral	Immoral	Neutral	total
**Group A1**	0	3	3	6
**Group A2**	2	6	1	9
**Group B1**	0	17	8	25
**Group B2**	4	19	5	28
**total**	**6**	**45**	**17**	**68**

*Note:* Each row represents the number of individual conditions (not participants) excluded. Conditions were excluded when participants failed the attention check question for that condition, indicating that the description may not have been read.

### 5.2. Effects of morality and humanness

Models 1, 1a and 1b examined the effects of Morality and Humanness on listeners’ emotional and aesthetic responses. As described in the ‘Analysis’ section, the random effects structure of Model 1 was adjusted to each dependent variable to address convergence issues. Accordingly, Model 1 (table 3) was fit to Moved, Beauty, Liking and Connected items, Model 1a (table 4) was fit to Sublimity, Vitality and Unease items, and Model 1b (table 5) was fit to Creativity.

**Table 3.** Linear mixed-effects model coefficients Model 1.

dependent variable	predictor	*b*	*SE*	95% *CI*	*df*	*t*	*p* (raw)	*p* (adj.)
**Moved**	**Intercept**	**54.85**	**3.47**	**[47.29, 62.41]**	**11.9**	**15.82**	**<0.001**	
	Moral	0.56	2.14	[−3.65, 4.76]	732.4	0.26	0.795	0.795
	**Immoral**	−**6.56**	**2.15**	**[**−**10.78,** −**2.33]**	**731.4**	−**3.05**	**0.002**	**0.003**
	AI	−4.32	3.10	[−10.41, 1.77]	764.0	−1.39	0.164	0.282
	Moral × AI	−0.21	3.04	[−6.18, 5.77]	737.8	−0.07	0.946	0.958
	Immoral × AI	2.23	3.13	[−3.91, 8.38]	738.3	0.71	0.476	0.750
	*Marginal/Conditional R² = 0.011/0.555*							
**Beauty**	**Intercept**	**67.00**	**3.00**	**[54.72, 79.28]**	**2.1**	**22.36**	**0.002**	
	Moral	−2.29	2.02	[−6.25, 1.68]	733.7	−1.13	0.258	0.795
	**Immoral**	−**8.28**	**2.03**	**[**−**12.27,** −**4.30]**	**732.4**	−**4.08**	**<0.001**	**<0.001**
	AI	−5.72	2.94	[−11.50, 0.05]	759.2	−1.95	0.052	0.282
	Moral × AI	3.01	2.87	[−2.63, 8.64]	738.7	1.05	0.295	0.958
	Immoral × AI	3.52	2.95	[−2.28, 9.31]	738.8	1.19	0.234	0.750
	*Marginal/Conditional R² = 0.014/0.552*							
**Liking**	**Intercept**	**64.78**	**2.81**	**[55.01, 74.55]**	**2.6**	**23.07**	**<0.001**	
	Moral	−1.50	2.09	[−5.61, 2.61]	705.9	−0.71	0.475	0.795
	**Immoral**	−**8.43**	**2.11**	**[**−**12.57,** −**4.28]**	**729.8**	−**3.99**	**<0.001**	**<0.001**
	AI	−4.12	3.05	[−10.10, 1.86]	764.4	−1.35	0.176	0.282
	Moral × AI	0.96	2.99	[−4.91, 6.82]	737.5	0.32	0.749	0.958
	Immoral × AI	2.45	3.08	[−3.59, 8.49]	743.4	0.80	0.426	0.750
	*Marginal/Conditional R² = 0.013/0.537*							
**Connected**	**Intercept**	**56.20**	**2.69**	**[48.01, 64.39]**	**3.3**	**20.91**	**<0.001**	
	Moral	1.46	2.08	[−2.61, 5.53]	732.5	0.70	0.482	0.795
	**Immoral**	−**7.26**	**2.09**	**[**−**11.36,** −**3.17]**	**732.4**	−**3.48**	**<0.001**	**0.001**
	AI	−2.34	3.20	[−8.61, 3.94]	710.8	−0.73	0.465	0.620
	Moral × AI	−1.31	2.95	[−7.11, 4.49]	738.5	−0.44	0.657	0.958
	Immoral × AI	0.75	3.04	[−5.22, 6.71]	737.9	0.25	0.806	0.918
	*Marginal/Conditional R² = 0.013/0.593*							

*Note*: *CI* = confidence interval; *df* = degrees of freedom calculated using Satterthwaite's method. Adjusted *p*-value = Benjamini–Hochberg adjusted *p*-value, corrected across the dependent variables separately. Model 1 was fit to Moved, Beauty, Liking and Connected items and includes a two-way interaction for Morality × Humanness and random intercepts for participants and songs nested within Vocalist Sex. Reference categories for intercepts: Morality = Neutral, Humanness = Human. Significant predictors are denoted in bold font.

The fixed effects for Models 1, 1a and 1b accounted for only a small portion of the variance across all dependent variables (marginal *R*² range: 0.003–0.014), with the addition of random effects accounting for more variance (conditional *R*² range: 0.473–0.648; see tables 3−5). Taken together, this suggests that our manipulations of Morality and Humanness accounted for a modest portion of the variance in listeners’ emotional and aesthetic responses. That said, after Benjamini–Hochberg corrections were applied, a significant main effect of Morality was observed for all dependent variables, except for Unease, *F*(2, 731.55) = 2.99, *p*_adj. = 0.051 (see figure 2). Contrary to what was predicted, no significant main effects of Humanness were found for any of the dependent variables (all *p*_adj. > 0.41). Similarly, no significant interactions between Morality and Humanness were observed (all *p*_adj. > 0.93), not supporting hypotheses 1 and 3.

**Figure 2.**
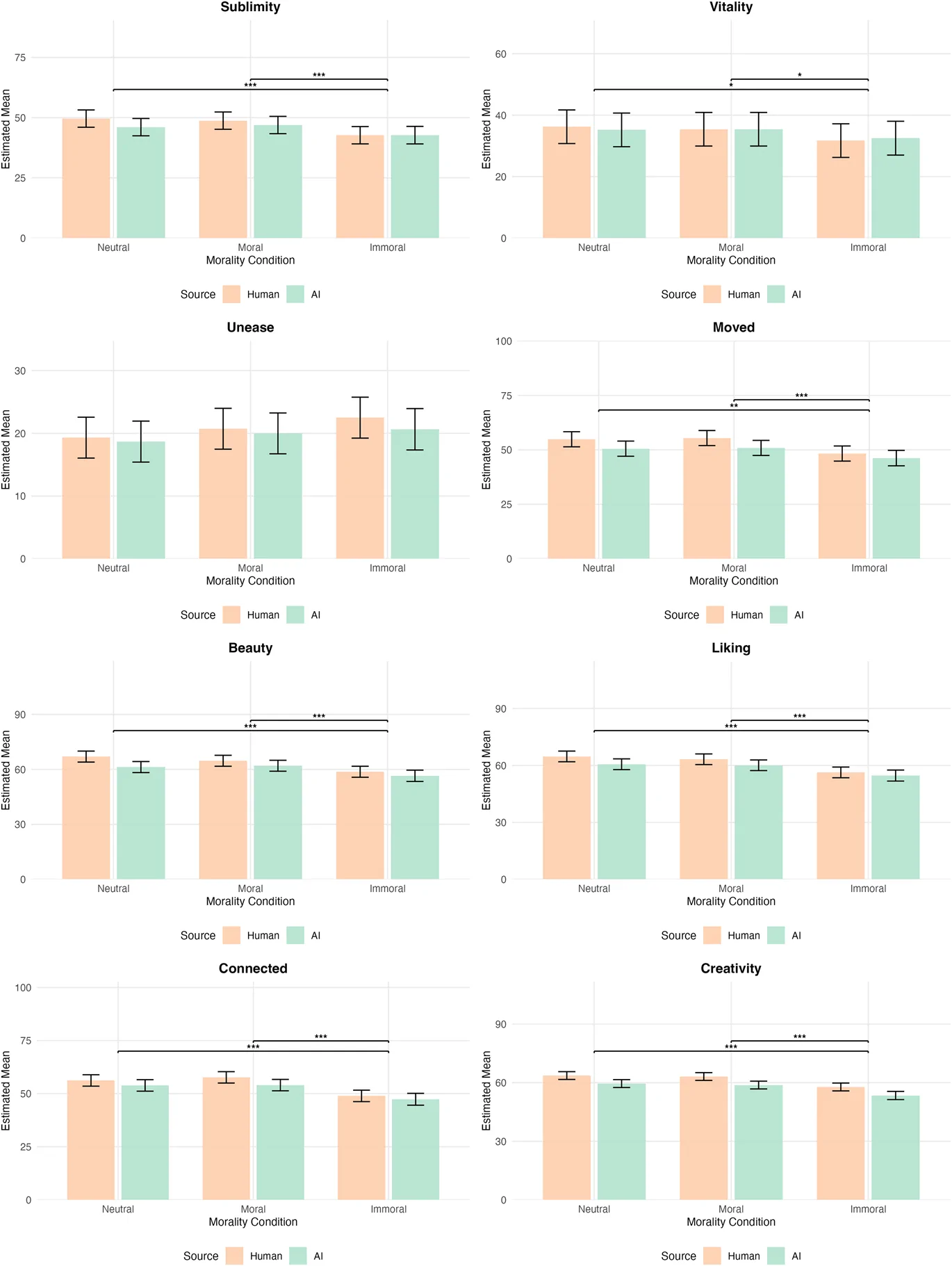
Effects of Morality and Humanness on emotional and aesthetic responses. *Note.* Estimated marginal means for each dependent variable, grouped by humanness, are shown across morality conditions. Error bars represent ±1 standard error. Significance brackets indicate Holm–Bonferroni corrected post hoc pairwise comparisons for the main effect of Morality (*p < 0.05, **p < 0.01, ***p < 0.001). The y-axis scales differ across each dependent variable as a result of different response ranges.

**Table 4.** Linear mixed-effects model coefficients Model 1a.

dependent variable	predictor	*b*	*SE*	95% *CI*	*df*	*t*	*p* (raw)	*p* (adj.)
**Sublimity**	**Intercept**	**49.61**	**3.59**	**[38.53, 60.69]**	**3.2**	**13.81**	**<0.001**	
	Moral	−0.85	1.58	[−3.95, 2.26]	731.8	−0.54	0.593	0.795
	**Immoral**	−**6.90**	**1.59**	**[**−**10.02,** −**3.78]**	**731.2**	−**4.34**	**<0.001**	**<0.001**
	AI	−3.54	2.42	[−8.29, 1.20]	717.5	−1.47	0.143	0.282
	Moral × AI	1.72	2.25	[−2.69, 6.13]	737.4	0.77	0.444	0.958
	Immoral × AI	3.57	2.31	[−0.97, 8.11]	737.7	1.54	0.123	0.750
	*Marginal/Conditional R² = 0.012/0.605*							
**Vitality**	**Intercept**	**36.24**	**5.47**	**[16.95, 55.53]**	**2.5**	**6.63**	**0.011**	
	Moral	−0.81	1.83	[−4.40, 2.77]	730.3	−0.45	0.656	0.795
	**Immoral**	−**4.51**	**1.83**	**[**−**8.11,** −**0.91]**	**729.9**	−**2.46**	**0.014**	**0.016**
	AI	−1.02	2.74	[−6.39, 4.35]	732.9	−0.37	0.709	0.741
	Moral × AI	1.02	2.59	[−4.07, 6.12]	736.2	0.39	0.694	0.958
	Immoral × AI	1.81	2.67	[−3.42, 7.05]	736.8	0.68	0.497	0.750
	*Marginal/Conditional R² = 0.003/0.606*							
**Unease**	**Intercept**	**19.31**	**3.26**	**[8.45, 30.17]**	**2.8**	**5.92**	**0.012**	
	Moral	1.42	1.45	[−1.42, 4.26]	721.9	0.98	0.327	0.795
	**Immoral**	**3.19**	**1.45**	**[0.34, 6.04]**	**721.9**	**2.20**	**0.028**	**0.028**
	AI	−0.63	1.91	[−4.38, 3.12]	850.7	−0.33	0.741	0.741
	Moral × AI	−0.11	2.05	[−4.14, 3.92]	728.6	−0.05	0.958	0.958
	Immoral × AI	−1.22	2.11	[−5.37, 2.92]	731.1	−0.58	0.563	0.750
	*Marginal/Conditional R² = 0.004/0.473*							

*Note*: *CI* = confidence interval; *df* = degrees of freedom calculated using Satterthwaite's method. Adjusted *p*-value = Benjamini–Hochberg adjusted *p*-value, corrected across the dependent variables separately. Model 1a was fit to Sublimity, Vitality and Unease items and includes a two-way interaction for Morality × Humanness and random intercepts for participants and songs. Reference categories for intercepts: Morality = Neutral, Humanness = Human. Significant predictors are denoted in bold font.

**Table 5.** Linear mixed-effects model coefficients Model 1b.

dependent variable	predictor	*b*	*SE*	95% *CI*	*df*	*t*	*p* (raw)	*p* (adj.)
**Creativity**	**Intercept**	**63.64**	**1.99**	**[59.73, 67.55]**	**642.6**	**31.95**	**<0.001**	
	Moral	−0.50	1.69	[−3.82, 2.83]	730.3	−0.29	0.770	0.795
	**Immoral**	−**5.85**	**1.71**	**[**−**9.20,** −**2.49]**	**730.2**	−**3.42**	**< 0.001**	**0.001**
	AI	−4.10	2.84	[−9.67, 1.46]	653.9	−1.45	0.148	0.282
	Moral × AI	−0.26	2.42	[−5.01, 4.49]	735.6	−0.11	0.915	0.958
	Immoral × AI	−0.26	2.50	[−5.16, 4.65]	736.0	−0.10	0.918	0.993
	*Marginal/Conditional R² = 0.014/0.648*							

*Note*: *CI* = confidence interval; *df* = degrees of freedom calculated using Satterthwaite's method. Adjusted *p*-value = Benjamini–Hochberg adjusted *p*-value, corrected across the dependent variables separately. Model 1b was fit to Creativity and includes a two-way interaction for Morality × Humanness and random intercepts for participants. Reference categories for intercepts: Morality = Neutral, Humanness = Human. Significant predictors are denoted in bold font.

### 5.3. Effects of morality and vocalist sex

Models 2 and 2a examined the effects of Morality and Vocalist Sex on listeners’ emotional and aesthetic responses, using only the data from the human musician participant groups. The random effects structure was simplified for each dependent variable as needed with Model 2 (table 6) fit to Sublimity, Vitality, Unease, Moved, Beauty and Connected and Model 2a (table 7) fit to Liking and Creativity.

**Table 6.** Linear mixed-effects model coefficients Model 2.

dependent variable	predictor	*b*	*SE*	95% *CI*	*df*	*t*	*p* (raw)	*p* (adj.)
**Sublimity**	**Intercept**	**48.91**	**4.10**	**[37.79, 60.04]**	**4.3**	**11.93**	**<0.001**	
	Moral	−2.38	2.30	[−6.91, 2.15]	382.3	−1.03	0.302	0.648
	**Immoral**	−**10.12**	**2.30**	**[**−**14.64,** −**5.59]**	**381.8**	−**4.40**	**< 0.001**	**< 0.001**
	Female	1.26	3.38	[−5.39, 7.91]	365.1	0.37	0.709	0.915
	Moral × Female	3.22	3.20	[−3.06, 9.51]	380.9	1.01	0.314	0.628
	Immoral × Female	6.48	3.22	[0.15, 12.82]	380.8	2.01	0.045	0.179
	*Marginal/Conditional R² = 0.026/0.594*							
**Vitality**	**Intercept**	**33.81**	**5.58**	**[16.86, 50.77]**	**3.3**	**6.06**	**0.007**	
	Moral	−0.91	2.53	[−5.88, 4.06]	380.3	−0.36	0.718	0.815
	**Immoral**	−**5.55**	**2.53**	**[**−**10.52,** −**0.58]**	**379.9**	−**2.20**	**0.029**	**0.033**
	Female	4.60	3.83	[−2.93, 12.12]	350.6	1.20	0.231	0.915
	Moral × Female	0.65	3.51	[−6.24, 7.55]	379.3	0.19	0.852	0.852
	Immoral × Female	2.45	3.54	[−4.51, 9.40]	379.1	0.69	0.490	0.653
	*Marginal/Conditional R² = 0.015/0.628*							
**Unease**	**Intercept**	**20.23**	**3.41**	**[10.78, 29.68]**	**4.0**	**5.94**	**0.004**	
	Moral	2.14	2.17	[−2.12, 6.40]	376.5	0.99	0.324	0.648
	Immoral	2.77	2.17	[−1.48, 7.03]	376.2	1.28	0.201	0.201
	Female	−1.71	2.76	[−7.13, 3.71]	434.9	−0.62	0.536	0.915
	Moral × Female	−1.66	3.01	[−7.58, 4.25]	374.8	−0.55	0.580	0.688
	Immoral × Female	0.61	3.03	[−5.35, 6.57]	374.9	0.20	0.841	0.865
	*Marginal/Conditional R² = 0.007/0.449*							
**Moved**	**Intercept**	**53.57**	**4.75**	**[41.48, 65.66]**	**5.2**	**11.28**	**<0.001**	
	Moral	−0.70	3.00	[−6.59, 5.19]	383.2	−0.23	0.815	0.815
	**Immoral**	−**9.51**	**2.99**	**[**−**15.39,** −**3.62]**	**382.6**	−**3.17**	**0.002**	**0.003**
	Female	2.74	4.30	[−5.72, 11.20]	375.5	0.64	0.525	0.915
	Moral × Female	2.17	4.16	[−6.01, 10.34]	381.5	0.52	0.602	0.688
	Immoral × Female	5.56	4.19	[−2.68, 13.80]	381.3	1.33	0.186	0.297
	*Marginal/Conditional R² = 0.019/0.567*							
**Beauty**	**Intercept**	**65.83**	**3.82**	**[57.22, 74.45]**	**9.3**	**17.22**	**< 0.001**	
	Moral	−6.07	2.85	[−11.68, −0.46]	384.2	−2.13	0.034	0.273
	**Immoral**	−**11.26**	**2.86**	**[**−**16.87,** −**5.64]**	**383.4**	−**3.94**	**< 0.001**	**< 0.001**
	Female	2.19	4.13	[−5.93, 10.31]	373.2	0.53	0.597	0.915
	Moral × Female	7.65	3.97	[−0.15, 15.45]	382.0	1.93	0.055	0.247
	Immoral × Female	6.09	4.00	[−1.77, 13.95]	381.7	1.52	0.129	0.297
	*Marginal/Conditional R² = 0.029/0.567*							
**Connected**	**Intercept**	**56.27**	**3.59**	**[48.93, 63.61]**	**28.9**	**15.68**	**<0.001**	
	Moral	−2.00	2.94	[−7.78, 3.79]	382.7	−0.68	0.498	0.664
	**Immoral**	−**10.20**	**2.95**	**[**−**16.00,** −**4.40]**	**384.0**	−**3.46**	**< 0.001**	**0.001**
	Female	−0.24	4.54	[−9.17, 8.69]	347.8	−0.05	0.958	0.958
	Moral × Female	6.91	4.10	[−1.15, 14.97]	382.5	1.69	0.093	0.247
	Immoral × Female	5.99	4.13	[−2.14, 14.12]	381.7	1.45	0.148	0.297
	*Marginal/Conditional R² = 0.02/0.608*							

*Note*: *CI* = confidence interval; *df* = degrees of freedom calculated using Satterthwaite's method. Adjusted *p*-value = Benjamini–Hochberg adjusted *p*-value, corrected across the dependent variables separately. Model 2 was fit to Sublimity, Vitality, Unease, Moved, Beauty and Connected items and includes a two−way interaction for Morality × Vocalist Sex and random intercepts for participants and songs. Reference categories: Morality = Neutral, Vocalist Sex = Male. Analysis restricted to human depictions only. Significant predictors are denoted in bold font.

The fixed effects for Models 2 and 2a explained a small proportion of variance (marginal *R*² range: 0.007–0.029), with random effects accounting for more variance in participants’ responses (conditional *R*² range: 0.449–0.671). Similar to Models 1, 1a and 1b, significant main effects of Morality were observed for all dependent variables after Benjamini–Hochberg corrections were applied, except for Unease, *F*(2, 376.32) = 2.02, *p*_adj. = 0.133. No significant main effects of Vocalist Sex were found for any of the dependent variables (all *p*_adj > 0.17). Similarly, no significant interactions between Morality and Vocalist Sex were observed (all *p*_adj > 0.35), not supporting hypothesis 4.

**Table 7.** Linear mixed-effects model coefficients Model 2a.

dependent variable	predictor	*b*	*SE*	95% *CI*	*df*	*t*	*p (raw)*	*p (adj.)*
**Liking**	**Intercept**	**64.26**	**3.03**	**[58.30, 70.21]**	**365.9**	**21.22**	**<0.001**	
	Moral	−5.06	2.88	[−10.72, 0.59]	384.5	−1.76	0.079	0.317
	**Immoral**	−**12.91**	**2.91**	**[**−**18.63,** −**7.18]**	**384.8**	−**4.43**	**<0.001**	**<0.001**
	Female	1.09	4.30	[−7.36, 9.53]	368.7	0.25	0.800	0.915
	Moral × Female	6.99	4.07	[−1.01, 14.99]	384.5	1.72	0.087	0.247
	Immoral × Female	8.83	4.11	[0.74, 16.91]	384.6	2.15	0.032	0.179
	*Marginal/Conditional R² = 0.02/0.608*							
**Creativity**	**Intercept**	**60.21**	**2.76**	**[54.78, 65.65]**	**315.7**	**21.80**	**<0.001**	
	Moral	−1.68	2.29	[−6.18, 2.82]	383.3	−0.74	0.462	0.664
	**Immoral**	−**5.57**	**2.32**	**[**−**10.13,** −**1.02]**	**383.4**	−**2.41**	**0.017**	**0.022**
	Female	6.88	3.92	[−0.83, 14.58]	317.9	1.76	0.080	0.640
	Moral × Female	2.32	3.24	[−4.05, 8.68]	383.3	0.72	0.475	0.688
	Immoral × Female	−0.56	3.27	[−6.98, 5.87]	383.2	−0.17	0.865	0.865
	*Marginal/Conditional R² = 0.02/0.608*							

*Note:* *CI* = confidence interval; *df* = degrees of freedom calculated using Satterthwaite's method. Adjusted *p*-value = Benjamini–Hochberg adjusted *p*-value, corrected across the dependent variables separately. Model 2a was fit to Liking and Creativity items and includes a two-way interaction for Morality × Vocalist Sex and random intercepts for participants. Reference categories: Morality = Neutral, Vocalist Sex = Male. Analysis restricted to human depictions only. Significant predictors are denoted in bold font.

### 5.4. Post hoc pairwise comparisons

Post hoc pairwise comparisons were conducted for Morality as this was the only predictor in which a significant main effect was observed in Models 1, 1a, 1b and Models 2 and 2a for each dependent variable, with the exception of Unease. Post hoc comparisons for Model 1 (table 8) compared the three morality conditions across both human and AI participant groups. Results from these comparisons indicate that when participants read immoral descriptions, their subsequent ratings for Sublimity, Vitality, Moved, Beauty, Liking, Connected and Creativity were significantly lower than when they read the neutral or moral descriptions. Contrary to what was predicted, comparisons between the neutral and moral conditions were non-significant in this analysis.

**Table 8.** Post hoc pairwise comparisons for morality Model 1.

dependent variable	contrast	estimate	*SE*	*df*	*t*	*p* (adj.)
**Sublimity**	Neutral–Moral	−0.01	1.12	737.7	−0.01	0.990
	**Neutral**–**Immoral**	**5.11**	**1.16**	**738.8**	**4.43**	**<0.001**
	**Moral**–**Immoral**	**5.13**	**1.15**	**742.6**	**4.47**	**<0.001**
**Vitality**	Neutral–Moral	0.30	1.30	737.9	0.23	0.816
	**Neutral**–**Immoral**	**3.60**	**1.33**	**739.3**	**2.70**	**0.021**
	**Moral**–**Immoral**	**3.30**	**1.32**	**743.4**	**2.49**	**0.026**
**Moved**	Neutral–Moral	−0.45	1.52	737.4	−0.30	0.766
	**Neutral**–**Immoral**	**5.44**	**1.57**	**738.9**	**3.48**	**0.001**
	**Moral**–**Immoral**	**5.89**	**1.55**	**742.7**	**3.80**	**<0.001**
**Beauty**	Neutral–Moral	0.78	1.43	738.2	0.55	0.585
	**Neutral**–**Immoral**	**6.53**	**1.48**	**739.3**	**4.42**	**<0.001**
	**Moral**–**Immoral**	**5.74**	**1.46**	**742.6**	**3.92**	**<0.001**
**Liking**	Neutral–Moral	1.02	1.50	738.7	0.68	0.498
	**Neutral**–**Immoral**	**7.20**	**1.54**	**742.3**	**4.67**	**<0.001**
	**Moral**–**Immoral**	**6.18**	**1.53**	**744.2**	**4.04**	**<0.001**
**Connected**	Neutral–Moral	−0.81	1.48	738.5	−0.55	0.586
	**Neutral**–**Immoral**	**6.89**	**1.52**	**738.6**	**4.53**	**< 0.001**
	**Moral**–**Immoral**	**7.70**	**1.51**	**740.9**	**5.10**	**< 0.001**
**Creativity**	Neutral–Moral	0.62	1.21	737.8	0.52	0.606
	**Neutral**–**Immoral**	**5.98**	**1.25**	**738.2**	**4.78**	**<0.001**
	**Moral**–**Immoral**	**5.35**	**1.24**	**742.1**	**4.30**	**<0.001**

*Note:* Post hoc pairwise comparisons for the main effect of Morality, conducted only for dependent variables with a significant FDR-adjusted morality effect (*p*_adj. < 0.05). Estimates represent mean differences between Morality conditions, averaged over the levels of Humanness. *df* = degrees of freedom (Kenward–Roger); *p*-values adjusted using the Holm–Bonferroni method. Unease was excluded as the main effect of Morality was not significant. Significant contrasts are denoted in bold font.

Post hoc comparisons for Model 2 (table 9) compared the three Morality conditions averaged over the levels of Vocalist Sex (Male and Female) for the Human participant groups only. Results from these post hoc comparisons largely replicated the post hoc comparisons from Model 1, with one notable difference; comparisons for Vitality were weaker in Model 2, with the Neutral–Immoral contrast retaining significance (*p_*adj. = 0.049), but not the Moral–Immoral contrast (*p*_adj. = 0.07). Additionally, the effect sizes (i.e. estimates) for post hoc comparisons for Model 2 were generally slightly larger than the effect sizes for post hoc comparisons for Model 1, with Creativity being an exception to this. Taken together, these post hoc comparisons for Models 1 and 2 offer partial support for hypothesis 2 and potentially suggest that there may be a stronger effect of Morality when the musician is believed to be Human compared to AI; however, the main and interaction effects related to Humanness failed to reach significance in both Models 1, 1a, 1b and Models 2 and 2a.

**Table 9.** Post hoc pairwise comparisons for morality Model 2.

dependent variable	contrast	estimate	*SE*	*df*	*t*	*p* (adj.)
**Sublimity**	Neutral–Moral	0.77	1.64	383.2	0.47	0.639
	**Neutral**–**Immoral**	**6.88**	**1.64**	**382.6**	**4.20**	**<0.001**
	**Moral**–**Immoral**	**6.11**	**1.61**	**382.9**	**3.79**	**<0.001**
**Vitality**	Neutral–Moral	0.59	1.80	382.6	0.33	0.745
	Neutral–Immoral	4.33	1.80	382.0	2.41	0.049
	Moral–Immoral	3.74	1.77	382.6	2.12	0.070
**Moved**	Neutral–Moral	−0.38	2.13	383.7	−0.18	0.857
	**Neutral**–**Immoral**	**6.73**	**2.13**	**383.0**	**3.16**	**0.003**
	**Moral**–**Immoral**	**7.11**	**2.10**	**383.1**	**3.39**	**0.002**
**Beauty**	Neutral–Moral	2.25	2.04	384.0	1.10	0.271
	**Neutral**–**Immoral**	**8.21**	**2.04**	**383.5**	**4.03**	**<0.001**
	**Moral**–**Immoral**	**5.97**	**2.00**	**383.2**	**2.98**	**0.006**
**Liking**	Neutral–Moral	1.57	2.03	383.5	0.77	0.441
	**Neutral**–**Immoral**	**8.50**	**2.06**	**383.6**	**4.13**	**<0.001**
	**Moral**–**Immoral**	**6.93**	**2.05**	**384.8**	**3.37**	**0.002**
**Connected**	Neutral–Moral	−1.46	2.11	380.5	−0.69	0.490
	**Neutral**–**Immoral**	**7.21**	**2.11**	**383.1**	**3.42**	**0.001**
	**Moral**–**Immoral**	**8.67**	**2.07**	**382.9**	**4.19**	**<0.001**
**Creativity**	Neutral–Moral	0.53	1.62	382.9	0.33	0.745
	**Neutral**–**Immoral**	**5.85**	**1.63**	**382.8**	**3.58**	**0.001**
	**Moral**–**Immoral**	**5.32**	**1.63**	**383.9**	**3.26**	**0.002**

*Note:* Post hoc pairwise comparisons for the main effect of Morality, conducted only for dependent variables with a significant FDR-adjusted Morality effect (*p*_adj. < 0.05). Estimates represent mean differences between Morality conditions, averaged over the levels of Vocalist Sex. *df* = Degrees of freedom (Kenward–Roger); *p*-values adjusted using the Holm–Bonferroni method. Analysis used Human participant groups only. Unease was excluded as the main effect of Morality was not significant. Significant contrasts are denoted in bold font.

## 6. Exploratory analyses

### 6.1. Empathic Concern

While a Kruskal–Wallis test revealed no significant differences across the four participant groups regarding trait empathy, a third linear mixed-effects model, Model 3, was fit to examine whether participants’ Trait Empathy moderated the effects of Morality and Humanness on response ratings as empathy has been associated with modulating the intensity of music-induced emotions [26,63] and experiences of being moved [54]. Using summed scores from participants’ responses to the Empathic Concern subscale of the IRI [51], Empathy was mean-centred and included as a continuous predictor. The same tiered approach of simplifying the random effects structure used for Models 1 and 2 was applied to Model 3 to address singular fit warnings. As a result, Model 3 was fit to Liking, Model 3a was fit to Sublimity, Vitality, Unease, Moved, Beauty and Connected, and Model 3b was fit to Creativity.

**Model 3. Maximum Random Structure: Liking**
DV ∼ Morality × Humanness × Empathy + (1|Participant_ID) + (1|Vocalist_sex/Music_ID)
**Model 3a. Unnested Random Structure: Sublimity, Vitality, Unease, Moved, Beauty and Connected**
DV ∼ Morality × Humanness × Empathy + (1|Participant_ID) + (1|Music_ID)
**Model 3b. Simplified Structure: Creativity**
DV ∼ Morality × Humanness × Empathy + (1|Participant_ID)

Results from Models 3, 3a and 3b indicated significant main effects of Empathy across seven of the eight dependent variables after Benjamini–Hochberg corrections with higher empathic concern consistently associated with more intense emotional and aesthetic responses to music, with the one exception being Unease (*p* = 0.05). The inclusion of Empathy as a fixed effect increased the Marginal *R*² for Models 3, 3a and 3b compared to Models 1 and 2 (Marginal *R*² range: 0.012–0.090), while the Conditional *R*² were similar to what was seen for Models 1 and 2 (Conditional *R*² range: 0.476–0.649). Empathy showed its largest associations for feelings of being Moved (*b* = 1.17), Connected (*b* = 1.73) and Beauty (*b* = 1.65). There were no significant interactions between Empathy and Morality (all *p_*adj. > 0.64), Empathy and Humanness (all *p_*adj. > 0.64), nor any significant three-way interactions between Morality × Humanness × Empathy (all *p_*adj. > 0.7). The main effects of Morality and Humanness were consistent with the results of the planned analyses. Full coefficient tables for Models 3, 3a and 3b are reported in tables S3, S4 and S5 in the supplementary materials.

### 6.2. Age

A second Kruskal–Wallis test indicated that participants’ Age significantly differed between Group A2 and Group B1. To account for this difference between participant groups and to evaluate the extent to which participants’ age moderated the effects of Morality, Humanness and their interaction on participants’ responses, a fourth linear mixed-effects model, Model 4 (shown below), was fit to participants’ emotional and aesthetic responses. Model 4 included fixed effects for Morality, Humanness and Age (mean-centred) and followed the same tiered approach for simplifying the random effects structure used in Models 1–3 to address singular fit warnings. As a result, Model 4 was fit to Vitality, Moved and Liking items, Model 4a was fit to Sublimity, Unease and Beauty items, and Model 4b was fit to Connected and Creativity items.

**Model 4. Maximum Random Structure: Vitality, Moved and Liking**
DV ∼ Morality × Humanness × Age + (1|Participant_ID) + (1|Vocalist_sex/Music_ID)
**Model 4a. Unnested Random Structure: Sublimity, Unease and Beauty**
DV ∼ Morality × Humanness × Age + (1|Participant_ID) + (1|Music_ID)
**Model 4b. Simplified Structure: Connected and Creativity**
DV ∼ Morality × Humanness × Age + (1|Participant_ID)

Results from Models 4, 4a and 4b indicated a significant main effect of Age across nearly all dependent variables after Benjamini–Hochberg corrections with exceptions for Unease (*p_*adj. = 0.265) and Connected (*p_*adj. = 0.107), with older participants associated with lower, less intense, emotional and aesthetic responses to the music. The inclusion of Age as a fixed effect roughly doubled the level of variance explained compared to Models 1 and 2 (Marginal *R*² range: 0.013–0.042); Conditional *R*² values remained similar (Conditional *R*² range: 0.476–0.649). Age showed its largest associations for Vitality (*b* = -0.51) and feelings of being Moved (*b* = -0.42). However, there were no significant interaction effects between Age and Morality (all *p_*adj. > 0.98), Age and Humanness (all *p_*adj. > 0.78), nor any significant three-way interaction between Morality × Humanness × Age (all *p_*adj. > 0.42). The main effects of Morality and Humanness in Model 4 were consistent with the results of Models 1 and 2, indicating that these effects did not vary significantly as a result of participant age. Full coefficient tables are reported in tables S6, S7 and S8 in the supplementary materials.

### 6.3. Failed deception

The final exploratory analysis excluded participants who correctly or closely guessed the true intention of the experiment prior to the disclosure of the debrief statement and then fitted the same models to the new, robust dataset. Of the total sample, 26 participants provided a genuine attempt to guess the study's true intention in response to the open-ended deception check question; the remaining participants either left the field blank or provided irrelevant responses (e.g. ‘No’, ‘N/A’, ‘Thank you’ or comments about the musical stimuli). Of these 26 responses, 16 were judged to have correctly or closely guessed the true intention of the study and were removed from this exploratory analysis (*n* = 4 participants removed from Group A1, *n* = 9 removed from Group A2, *n* = 2 removed from Group B1 and *n* = 1 removed from Group B2). The results from this exploratory analysis were largely consistent with the results from the planned analyses, with one notable exception. For Vitality ratings, the significance of the post hoc pairwise comparisons between the Moral–Immoral conditions (Estimate *=* 3.07, *SE =* 1.36, *t*(711) *=* 2.25, *p_*adj. *=* 0.058) and Neutral–Immoral conditions (Estimate *=* 3.21, *SE =* 1.37, *t*(707) *=* 2.34, *p_*adj. *=* 0.058) did not retain statistical significance.

## 7. Discussion

The present experiment evaluated the extent to which a musician's Humanness and Morality influenced listeners’ emotional and aesthetic responses to their songs. In light of the predictions, the findings from the present experiment did not support the prediction that listeners’ emotional and aesthetic responses differ when the music is believed to be created by a generative AI model compared to a Human (H1). Partial support was found for the prediction that Moral depictions would lead to higher emotional and aesthetic response ratings (relative to Neutral), while Immoral depictions would lead to lower ratings (H2): for nearly all dependent variables, Immoral depictions led to significantly lower (i.e. more negative) ratings, with the exception of Unease and Vitality which did not retain significance for Moral–Immoral and Neutral–Immoral comparisons once the participants who failed the deception check were removed. However, no significant differences were observed between the Neutral and Moral descriptions for any of the dependent variables. No support was found for the hypothesized moderating effect of the Humanness of the song's creator on the Moral depictions (H3). Lastly, there were no significant main effects of Vocalist Sex, nor interactions between Vocalist Sex and Moral depictions of the Human musicians; therefore, H4 was not supported.

These findings demonstrate that, similarly to empathy for pain and the aesthetic valuation of visual art, the emotional and aesthetic responses evoked by music are modulated by information depicting musicians as Immoral. Specifically, listeners’ ratings of felt emotions of Sublimity, feelings of being Moved, Connected and aesthetic appraisals of Beauty, Liking and Creativity of the songs were affected in a negative way—with positive emotions and aesthetic responses becoming weaker—when descriptions portraying Immoral behaviour or actions were provided about the musician or the AI model. This significant effect persisted for dependent variables: Sublimity, Moved, Beauty, Liking, Connected and Creativity after excluding the participants who had correctly (or nearly correctly) guessed the true aim of the study, suggesting that the effect was not owing to demand characteristics.

Conversely, descriptions of Moral behaviour did not significantly increase aesthetic responses or felt emotions such as feelings of being moved relative to Neutral descriptions, contrary to what was predicted on the basis of previous studies and theoretical frameworks of being moved (e.g. [20,24,25,21]). It may be that prosocial intentions or moral connotations need to be expressed by the music itself—either through lyrics or musical features (see [64])—in order to effectively modulate listeners’ emotional and aesthetic responses. However, future studies are needed to systematically explore this possibility. Another possible explanation is that, since the tendency to appreciate beauty and aesthetic experiences has been shown to be perceived as a sign of increased prosociality and sensitivity [65], just being identified as a musician, without any additional information about moral behaviour, may already lead one to be interpreted as having good moral character.

Alternatively, these findings may reflect a more general social bias towards negative information [66]. The positive–negative asymmetry effect [67] argues that negative information about events, people and experiences carries a greater weight than positive information of equivalent importance. Rozin and Royzman's discussion of negative contagion [68] further builds on this, suggesting that negative qualities transfer to associated events, objects and people more readily than positive qualities, and, perhaps more crucially, that there is no equivalent ‘positive contamination’. This suggests that positive qualities are substantially weaker at elevating an associated event, object or person than negative qualities are at diminishing associated events, objects, people etc. [68].

For the exploratory analyses, both participants’ Trait Empathy and Age were observed as having significant main effects on participants’ response ratings. However, neither Trait Empathy nor Age interacted with Morality or Humanness. Trait empathic concern significantly predicted the intensity of emotional and aesthetic responses (with the exception of Unease), aligning with previous studies associating empathic concern with the enjoyment of sad and tender music and the intensity of feeling moved by music [63,69]. Although previous studies have linked empathic concern with an increased sensitivity to injustice for others [55], the present study did not find support for individuals higher in empathic concern being more strongly influenced by moral information about musicians. Increasing Age, on the other hand, had a significantly negative effect on the intensity of emotional and aesthetic responses (with the exceptions of Unease and Connected); with an increase in Age associated with lower, less intense, responses to the music, particularly concerning Vitality and being Moved, which may be seen as high-arousal emotional responses. This age-related dampening effect is consistent with previous research by Keil and Freund [70], who observed a significant decrease in the intensity of positive arousal ratings of pictures as participant age increased, though further research is needed to understand this effect of Age more clearly. The effect of Age observed in the present study may also be at least partly related to age-related differences in musical taste [e.g. 71].

With regard to the non-significant interaction between Age and Humanness, while a previous study [72] found that older adults tend to be more sceptical and less trusting of AI-generated advice, research on age-related differences in the perception of generative AI more broadly remains scarce. As a result, it is unclear to what extent age may moderate emotional and aesthetic responses to music portrayed as AI-generated specifically. However, it should also be noted that participants in this study were not recruited based on age, and Age was included as an exploratory analysis to account for a significant age difference observed between participant groups.

Taken together, these findings highlight the role of social cognition and social processes in music listening and musical engagement. Music is imbued with signs of human expression and agency at multiple levels, to the extent that music can sometimes evoke pseudo-social experiences such as providing company, consolation or empathy [73]. The present study extends these previous findings by suggesting that aesthetic and emotional engagement with music is grounded in social cognition and shaped by beliefs about the creator, particularly those regarding the creator's morality. Present findings suggest that, at least in the case of listening to unfamiliar music, one cannot entirely separate the art from the artist.

## 8. Limitations and future directions

The present study failed to find significant differences in emotional and aesthetic responses between the conditions where the music was portrayed as AI-generated compared to Human-created. Similarly, there was no significant interaction effect between Humanness and Morality. However, it is possible that the lack of significant effects is at least partly owing to the experimental design, where Humanness was implemented as a between-subjects factor and Morality was implemented as a within-subjects factor. This design choice was made in the present study to strengthen the believability of the manipulations and to limit the number of stimuli presented to each participant (in order not to reveal the real aim of the study). A previous study investigating the effect of composer identity (Human vs. AI) on aesthetic evaluations found stronger effects in the experiments using within-subjects designs compared to the experiments using between-subjects designs [47]. It is likely that, besides controlling for some of the interindividual variance, a within-subjects design makes the contrast between conditions—whether Human vs. AI or Moral vs. Immoral—more salient. It may also be that the current study was underpowered with respect to the between-subjects variable, as some effects of Humanness were approaching the significance threshold in the raw, uncorrected *p*-values. Another explanation for this may be that the experimental conditions did not successfully isolate Humanness as a variable, meaning that participants in the AI groups may have been evaluating the Morality of the humans who created the generative AI models, rather than the AI model itself, effectively resulting in comparing two categories of human creators rather than Human vs. AI. Additionally, the design of the present study cannot fully disentangle whether the effects of Morality reflect moral judgement specifically or a more general valence-priming effect, whereby reading a negative description, in turn, negatively affects subsequent ratings. Nevertheless, more research is needed to understand how listeners engage with (purportedly) AI-generated music. Future studies could address the present limitations by including conditions that more clearly distance the humans who created the AI from the AI model creating the music and by incorporating additional conditions that attempt to disentangle moral judgement from the more general valence-priming effects by using descriptions that include emotionally negative but morally neutral control descriptions.

While the exploratory analyses evaluating participants’ Trait Empathy and Age as potential moderators of the effects of Morality and Humanness on emotional and aesthetic responses revealed significant main effects for both Empathy and Age, neither analysis indicated any significant two- or three-way interactions involving Morality or Humanness. These null findings should be interpreted with caution, however, as it is likely that the present study did not have the statistical power to detect a three-way interaction.

Further limitations concern the musical stimuli and descriptions used in the present study. Regarding the music, we only used a small selection of musical stimuli from the singer-songwriter/pop genre, which may not fully represent the diversity of emotional and aesthetic experiences across genres, musical structures or performers. Regarding the morality descriptions, it is possible that the believability and effectiveness of the descriptions may have changed when paired with music in the main experiment. While the most believable, effective descriptions were selected from the first pilot study, these descriptions were evaluated in isolation, not in the presence of music. Furthermore, the moral manipulations that we employed in the main study were exclusively derived from the Care foundation of the Moral Foundations Theory [7]. This means that we cannot determine if the observed effects of moral information generalize across other core moral dimensions, such as Fairness, Loyalty, Authority or Sanctity/Purity. It is plausible that moral transgressions related to Loyalty (e.g. an artist betraying a creative partner) or Fairness (e.g. an artist plagiarizing another artist's work) might elicit differing effects on emotional and aesthetic responses compared to breaches of the Care foundation. Future studies are therefore needed to explore the full range of moral foundations as well as other aspects of Morality and to incorporate more varied and extensive selections of musical materials.

Future studies should also employ methods that are able to isolate the underlying mechanisms of the observed effects of moral information. For example, if information about a musician's immoral behaviour modulates emotion induction via empathy-related mechanisms (as speculated), such manipulations should be associated with reduced activation of emotion-sharing and mentalizing networks in the listener's brain (e.g. [45,18]). The present study has pointed towards the types of manipulations and stimuli that could be fruitful in pinpointing the underlying mechanisms of morality-evoked affective modulations in future neuroimaging work.

## 9. Conclusions

This study demonstrated that information about a musician's morality influences listeners’ emotional and aesthetic responses to their music. This significant effect persisted even after excluding participants who correctly guessed the true aim of the study. In line with previous evidence from the domain of visual art, we found that, compared with neutral information, information about a musician's Immoral behaviour can negatively affect the listener's emotional experience and aesthetic appreciation of their music. At the same time, the present findings indicated that listener's emotional and aesthetic responses did not significantly differ whether they were informed that the music was created by a human or generated by an AI model. Although we cannot determine whether this finding is a result of methodological choices made in this experiment or whether the wider audience is becoming more used to AI-generated music because of the rapid development of generative AI, it is important to continue investigating audience reception of music generated by AI. This topic has great societal relevance, as artists and creators in all domains are increasingly concerned about the potential future of their jobs in light of the proliferation of generative AI. Taken together, this study has increased our current understanding of the role of extra-musical information in emotions and aesthetic responses evoked by music, highlighting the role of socio-cognitive processes in musical engagement.

## Supplementary Material



## Data Availability

The data and R code for conducting all analyses reported in this study are publicly available on the project's page via Open Science Framework [74]. Supplementary material is available online [75].

## Footnotes

^1^
Equal numbers of male and female participants were recruited; however, the final male/female split is uneven due to differential dropout rates, as Prolific permits participants to return the survey without completing it.

^2^
A student intern selected 13 songs from artlist.io based on these four criteria. The primary author then chose the subset of eight songs from this selection pool to be used in the pilot experiment.

^3^
Octave displacement was implemented when necessary. For example, if the song was originally sung by a male vocalist in an octave that was unnaturally low for a female voice to produce, the melody was shifted up 12 semitones.

^4^
‘Feeling moved’ is typically included in the Sublimity Index in the GEMS-9. However, we treated this as a separate, independent item.
