# Supplementary material for: Moral echoes: do morality and humanness affect emotional and aesthetic responses to music?
Source: R Soc Open Sci. 2026 Oct 21;13(10):252167. doi: 10.1098/rsos.252167 (PMC13682890; doi:10.1098/rsos.252167)
Supplement: Supplementary file 1 [file rsos.252167.zip › rsos.252167/rsos252167_si_001.docx]

**Table S1. Type III ANOVA Table: Humanness × Vocalist Sex**

| Dependent Variable | Effect | Sum Sq | Mean Sq | *NumDF* | *DenDF* | *F* | Raw *p*-value | Adjusted *p*-value |
| --- | --- | --- | --- | --- | --- | --- | --- | --- |
| **Sublimity Index** | Humanness | 159.9 | 159.90 | 1 | 393.37 | 0.65 | .420 | .529 |
|  | Vocalist_sex | 492.3 | 492.26 | 1 | 393.39 | 2.00 | .158 | .210 |
|  | Humanness × Vocalist_sex | 135.7 | 135.73 | 1 | 393.36 | 0.55 | .458 | .798 |
| **Vitality Index** | Humanness | 0.0 | 0.03 | 1 | 391.29 | 0.00 | .992 | .992 |
|  | Vocalist_sex | 2603.7 | 2603.73 | 1 | 391.32 | 8.14 | .005 | .036 |
|  | Humanness × Vocalist_sex | 48.1 | 48.09 | 1 | 391.29 | 0.15 | .698 | .798 |
| **Unease Index** | Humanness | 108.1 | 108.09 | 1 | 380.87 | 0.54 | .463 | .529 |
|  | Vocalist_sex | 165.9 | 165.90 | 1 | 380.90 | 0.83 | .363 | .363 |
|  | Humanness × Vocalist_sex | 41.9 | 41.92 | 1 | 380.87 | 0.21 | .648 | .798 |
| **Moved** | Humanness | 831.4 | 831.38 | 1 | 393.84 | 1.87 | .173 | .460 |
|  | Vocalist_sex | 1006.0 | 1006.03 | 1 | 393.86 | 2.26 | .134 | .210 |
|  | Humanness × Vocalist_sex | 130.2 | 130.24 | 1 | 393.83 | 0.29 | .589 | .798 |
| **Beauty** | Humanness | 767.9 | 767.94 | 1 | 394.11 | 1.92 | .167 | .460 |
|  | Vocalist_sex | 1614.9 | 1614.93 | 1 | 394.14 | 4.04 | .045 | .181 |
|  | Humanness × Vocalist_sex | 222.5 | 222.48 | 1 | 394.11 | 0.56 | .456 | .798 |
| **Liking** | Humanness | 543.9 | 543.86 | 1 | 395.46 | 1.24 | .266 | .529 |
|  | Vocalist_sex | 1308.7 | 1308.72 | 1 | 395.47 | 2.98 | .085 | .210 |
|  | Humanness × Vocalist_sex | 255.8 | 255.82 | 1 | 395.46 | 0.58 | .446 | .798 |
| **Connected** | Humanness | 307.8 | 307.79 | 1 | 393.53 | 0.72 | .397 | .529 |
|  | Vocalist_sex | 903.4 | 903.36 | 1 | 393.55 | 2.11 | .147 | .210 |
|  | Humanness × Vocalist_sex | 0.5 | 0.49 | 1 | 393.53 | 0.00 | .973 | .973 |
| **Creative** | Humanness | 785.3 | 785.26 | 1 | 392.61 | 2.72 | .100 | .460 |
|  | Vocalist_sex | 465.9 | 465.90 | 1 | 392.61 | 1.62 | .205 | .234 |
|  | Humanness × Vocalist_sex | 887.9 | 887.92 | 1 | 392.61 | 3.08 | .080 | .641 |
| ***Note.*** Type III Analysis of Variance with Satterthwaite's method. *NumDF* = numerator degrees of freedom; *DenDF* = denominator degrees of freedom. Adjusted p-value = Benjamini-Hochberg adjusted, corrected across the eight dependent variables separately. Analysis includes data from all four participant groups. | | | | | | | | |

**Table S2. Type III ANOVA Table: Morality × Humanness × Vocalist Sex**

| Dependent Variable | Effect | Sum Sq | Mean Sq | *NumDF* | *DenDF* | *F* | Raw *p*-value | Adjusted *p*-value |
| --- | --- | --- | --- | --- | --- | --- | --- | --- |
| **Sublimity Index** | Morality | 7472.0 | 3735.98 | 2 | 738.73 | 13.96 | < .001 | < .001 |
|  | Humanness | 200.1 | 200.12 | 1 | 395.38 | 0.75 | .388 | .517 |
|  | Vocalist_sex | 669.6 | 669.59 | 1 | 395.38 | 2.50 | .115 | .170 |
|  | Morality × Humanness | 1549.9 | 774.97 | 2 | 738.73 | 2.89 | .056 | .447 |
|  | Morality × Vocalist_sex | 1245.7 | 622.86 | 2 | 738.73 | 2.33 | .098 | .280 |
|  | Humanness × Vocalist_sex | 129.9 | 129.92 | 1 | 395.38 | 0.49 | .486 | .763 |
|  | Morality × Humanness × Vocalist_sex | 656.1 | 328.05 | 2 | 738.73 | 1.23 | .294 | .621 |
| **Vitality Index** | Morality | 2904.6 | 1452.30 | 2 | 733.08 | 4.57 | .011 | .012 |
|  | Humanness | 0.2 | 0.21 | 1 | 392.29 | 0.00 | .979 | .979 |
|  | Vocalist_sex | 2757.4 | 2757.35 | 1 | 392.32 | 8.68 | .003 | .027 |
|  | Morality × Humanness | 141.6 | 70.78 | 2 | 733.37 | 0.22 | .800 | .940 |
|  | Morality × Vocalist_sex | 1000.8 | 500.41 | 2 | 732.98 | 1.58 | .208 | .415 |
|  | Humanness × Vocalist_sex | 64.1 | 64.06 | 1 | 392.29 | 0.20 | .654 | .763 |
|  | Morality × Humanness × Vocalist_sex | 497.4 | 248.71 | 2 | 732.99 | 0.78 | .457 | .621 |
| **Unease Index** | Morality | 1204.5 | 602.25 | 2 | 727.57 | 3.01 | .050 | .050 |
|  | Humanness | 105.3 | 105.33 | 1 | 381.41 | 0.53 | .469 | .535 |
|  | Vocalist_sex | 179.2 | 179.22 | 1 | 381.45 | 0.90 | .345 | .345 |
|  | Morality × Humanness | 78.3 | 39.15 | 2 | 727.87 | 0.20 | .822 | .940 |
|  | Morality × Vocalist_sex | 18.2 | 9.11 | 2 | 727.36 | 0.05 | .955 | .955 |
|  | Humanness × Vocalist_sex | 37.0 | 37.01 | 1 | 381.41 | 0.18 | .667 | .763 |
|  | Morality × Humanness × Vocalist_sex | 291.9 | 145.95 | 2 | 727.38 | 0.73 | .483 | .621 |
| **Moved** | Morality | 7733.7 | 3866.85 | 2 | 736.86 | 8.86 | < .001 | < .001 |
|  | Humanness | 875.5 | 875.50 | 1 | 395.02 | 2.01 | .157 | .420 |
|  | Vocalist_sex | 1119.0 | 1119.04 | 1 | 395.05 | 2.56 | .110 | .170 |
|  | Morality × Humanness | 312.3 | 156.14 | 2 | 737.17 | 0.36 | .699 | .940 |
|  | Morality × Vocalist_sex | 1973.4 | 986.68 | 2 | 736.65 | 2.26 | .105 | .280 |
|  | Humanness × Vocalist_sex | 100.9 | 100.91 | 1 | 395.02 | 0.23 | .631 | .763 |
|  | Morality × Humanness × Vocalist_sex | 538.0 | 269.00 | 2 | 736.65 | 0.62 | .540 | .621 |
| **Beauty** | Morality | 8932.7 | 4466.35 | 2 | 736.62 | 11.53 | < .001 | < .001 |
|  | Humanness | 808.1 | 808.13 | 1 | 394.78 | 2.09 | .149 | .420 |
|  | Vocalist_sex | 1682.1 | 1682.06 | 1 | 394.80 | 4.34 | .038 | .151 |
|  | Morality × Humanness | 625.1 | 312.53 | 2 | 736.97 | 0.81 | .447 | .940 |
|  | Morality × Vocalist_sex | 579.7 | 289.87 | 2 | 736.17 | 0.75 | .473 | .631 |
|  | Humanness × Vocalist_sex | 192.0 | 192.01 | 1 | 394.77 | 0.50 | .482 | .763 |
|  | Morality × Humanness × Vocalist_sex | 2134.4 | 1067.18 | 2 | 736.16 | 2.76 | .064 | .373 |
| **Liking** | Morality | 10845.2 | 5422.58 | 2 | 740.41 | 12.75 | < .001 | < .001 |
|  | Humanness | 595.5 | 595.51 | 1 | 396.58 | 1.40 | .237 | .475 |
|  | Vocalist_sex | 1353.6 | 1353.60 | 1 | 396.58 | 3.18 | .075 | .170 |
|  | Morality × Humanness | 289.9 | 144.96 | 2 | 740.41 | 0.34 | .711 | .940 |
|  | Morality × Vocalist_sex | 1978.5 | 989.26 | 2 | 740.41 | 2.33 | .098 | .280 |
|  | Humanness × Vocalist_sex | 241.9 | 241.89 | 1 | 396.58 | 0.57 | .451 | .763 |
|  | Morality × Humanness × Vocalist_sex | 405.4 | 202.70 | 2 | 740.41 | 0.48 | .621 | .621 |
| **Connected** | Morality | 12672.1 | 6336.06 | 2 | 735.23 | 15.36 | < .001 | < .001 |
|  | Humanness | 349.0 | 349.01 | 1 | 394.49 | 0.85 | .358 | .517 |
|  | Vocalist_sex | 962.7 | 962.67 | 1 | 394.51 | 2.33 | .127 | .170 |
|  | Morality × Humanness | 188.5 | 94.24 | 2 | 735.51 | 0.23 | .796 | .940 |
|  | Morality × Vocalist_sex | 791.2 | 395.62 | 2 | 734.41 | 0.96 | .384 | .614 |
|  | Humanness × Vocalist_sex | 0.2 | 0.17 | 1 | 394.49 | 0.00 | .984 | .984 |
|  | Morality × Humanness × Vocalist_sex | 1963.3 | 981.64 | 2 | 734.37 | 2.38 | .093 | .373 |
| **Creative** | Morality | 7638.4 | 3819.21 | 2 | 732.93 | 13.59 | < .001 | < .001 |
|  | Humanness | 849.4 | 849.36 | 1 | 393.43 | 3.02 | .083 | .420 |
|  | Vocalist_sex | 482.5 | 482.48 | 1 | 393.43 | 1.72 | .191 | .218 |
|  | Morality × Humanness | 3.6 | 1.81 | 2 | 732.93 | 0.01 | .994 | .994 |
|  | Morality × Vocalist_sex | 41.9 | 20.94 | 2 | 732.93 | 0.07 | .928 | .955 |
|  | Humanness × Vocalist_sex | 840.7 | 840.73 | 1 | 393.43 | 2.99 | .084 | .676 |
|  | Morality × Humanness × Vocalist_sex | 308.0 | 153.98 | 2 | 732.93 | 0.55 | .578 | .621 |
| ***Note.*** Type III Analysis of Variance with Satterthwaite's method. *NumDF* = numerator degrees of freedom; *DenDF* = denominator degrees of freedom. Adjusted p-value = Benjamini-Hochberg adjusted, corrected across the eight dependent variables separately. Analysis includes data from all four participant groups. | | | | | | | | |

**Table S3. Linear Mixed-Effects Model Coefficients Model 3**

| *Dependent Variable* | *Predictor* | *b* | *SE* | *95% CI* | *df* | *t* | *p*  *(raw)* | *p*  *(adj.)* |
| --- | --- | --- | --- | --- | --- | --- | --- | --- |
| **Liking** | **Intercept** | **64.59** | **2.22** | **[59.05, 70.12]** | **5.6** | **29.11** | **< .001** |  |
|  | Moral | -1.56 | 2.10 | [-5.67, 2.56] | 704.6 | -0.74 | .458 | .746 |
|  | **Immoral** | **-8.50** | **2.12** | **[-12.66, -4.35]** | **727.1** | **-4.02** | **< .001** | **< .001** |
|  | AI | -3.57 | 2.95 | [-9.36, 2.22] | 793.2 | -1.21 | .226 | .362 |
|  | **Empathy** | **1.58** | **0.40** | **[0.80, 2.37]** | **781.0** | **3.95** | **< .001** | **< .001** |
|  | Moral × AI | 1.12 | 2.99 | [-4.75, 7.00] | 734.5 | 0.37 | .708 | .975 |
|  | Immoral × AI | 2.43 | 3.08 | [-3.62, 8.49] | 740.7 | 0.79 | .430 | .746 |
|  | Moral × Empathy | 0.32 | 0.41 | [-0.48, 1.12] | 731.2 | 0.79 | .430 | .747 |
|  | Immoral × Empathy | 0.05 | 0.41 | [-0.75, 0.85] | 731.5 | 0.12 | .904 | .963 |
|  | AI × Empathy | -0.16 | 0.59 | [-1.31, 0.99] | 802.4 | -0.28 | .780 | .780 |
|  | Moral × AI × Empathy | -0.12 | 0.59 | [-1.28, 1.04] | 739.0 | -0.20 | .839 | .895 |
|  | Immoral × AI × Empathy | -0.26 | 0.61 | [-1.45, 0.94] | 736.9 | -0.42 | .674 | .899 |
| ***Note.*** CI = confidence interval; df = degrees of freedom calculated using Satterthwaite's method. Adjusted p-value = Benjamini-Hochberg adjusted p-value, corrected across all dependent variables separately. Model 3 includes random intercepts for participants and songs nested within vocalist sex and fixed effects for Morality, Humanness, and Empathy, a two-way interaction between Morality × Humanness, Morality × Empathy, Humanness × Empathy, and a three-way interaction between Morality × Humanness × Empathy. Empathy is mean-centered. Reference categories: Morality = Neutral, Humanness = Human. | | | | | | | | |

**Table S4. Linear Mixed-Effects Model Coefficients Model 3a**

| *Dependent Variable* | *Predictor* | *b* | *SE* | *95% CI* | *df* | *t* | *p*  *(raw)* | *p*  *(adj.)* |
| --- | --- | --- | --- | --- | --- | --- | --- | --- |
| **Sublimity Index** | **Intercept** | **49.48** | **3.58** | **[38.37, 60.60]** | **3.1** | **13.84** | **< .001** |  |
|  | Moral | -0.87 | 1.58 | [-3.97, 2.24] | 727.6 | -0.55 | .584 | .746 |
|  | **Immoral** | **-6.86** | **1.59** | **[-9.98, -3.74]** | **727.1** | **-4.31** | **< .001** | **< .001** |
|  | AI | -3.25 | 2.38 | [-7.92, 1.42] | 727.0 | -1.37 | .172 | .362 |
|  | **Empathy** | **1.19** | **0.32** | **[0.55, 1.82]** | **710.6** | **3.68** | **< .001** | **< .001** |
|  | Moral × AI | 1.83 | 2.25 | [-2.59, 6.24] | 733.0 | 0.81 | .416 | .975 |
|  | Immoral × AI | 3.52 | 2.31 | [-1.02, 8.06] | 733.7 | 1.52 | .129 | .746 |
|  | Moral × Empathy | -0.09 | 0.30 | [-0.68, 0.51] | 725.6 | -0.29 | .773 | .883 |
|  | Immoral × Empathy | -0.46 | 0.31 | [-1.06, 0.14] | 726.4 | -1.49 | .136 | .543 |
|  | AI × Empathy | -0.58 | 0.47 | [-1.51, 0.34] | 733.1 | -1.24 | .216 | .576 |
|  | Moral × AI × Empathy | 0.36 | 0.44 | [-0.51, 1.23] | 734.5 | 0.81 | .416 | .832 |
|  | Immoral × AI × Empathy | 0.71 | 0.45 | [-0.18, 1.60] | 731.3 | 1.56 | .120 | .401 |
| **Vitality Index** | **Intercept** | **36.14** | **5.48** | **[16.76, 55.52]** | **2.5** | **6.60** | **.012** |  |
|  | Moral | -0.82 | 1.82 | [-4.40, 2.76] | 725.6 | -0.45 | .653 | .746 |
|  | **Immoral** | **-4.40** | **1.83** | **[-8.00, -0.80]** | **725.2** | **-2.40** | **.017** | **.019** |
|  | AI | -0.83 | 2.72 | [-6.17, 4.52] | 731.0 | -0.30 | .761 | .761 |
|  | **Empathy** | **1.13** | **0.37** | **[0.40, 1.85]** | **714.5** | **3.05** | **.002** | **.003** |
|  | Moral × AI | 1.09 | 2.59 | [-4.00, 6.18] | 731.1 | 0.42 | .675 | .975 |
|  | Immoral × AI | 1.69 | 2.67 | [-3.54, 6.93] | 732.0 | 0.64 | .525 | .746 |
|  | Moral × Empathy | -0.35 | 0.35 | [-1.03, 0.34] | 723.8 | -0.99 | .324 | .747 |
|  | Immoral × Empathy | -0.82 | 0.35 | [-1.52, -0.13] | 724.7 | -2.34 | .019 | .156 |
|  | AI × Empathy | -0.82 | 0.54 | [-1.88, 0.24] | 737.2 | -1.51 | .131 | .525 |
|  | Moral × AI × Empathy | 0.56 | 0.51 | [-0.45, 1.56] | 732.9 | 1.09 | .276 | .832 |
|  | Immoral × AI × Empathy | 0.82 | 0.52 | [-0.21, 1.85] | 729.7 | 1.57 | .117 | .401 |
| **Unease Index** | **Intercept** | **19.35** | **3.27** | **[8.44, 30.26]** | **2.8** | **5.92** | **.012** |  |
|  | Moral | 1.41 | 1.45 | [-1.43, 4.25] | 718.0 | 0.98 | .329 | .746 |
|  | **Immoral** | **3.20** | **1.45** | **[0.35, 6.06]** | **718.1** | **2.20** | **.028** | **.028** |
|  | AI | -0.74 | 1.91 | [-4.48, 3.01] | 848.0 | -0.39 | .700 | .761 |
|  | Empathy | -0.44 | 0.26 | [-0.94, 0.07] | 831.5 | -1.69 | .092 | .092 |
|  | Moral × AI | -0.20 | 2.05 | [-4.23, 3.83] | 724.3 | -0.10 | .922 | .975 |
|  | Immoral × AI | -1.23 | 2.11 | [-5.38, 2.91] | 727.1 | -0.58 | .559 | .746 |
|  | Moral × Empathy | 0.18 | 0.28 | [-0.37, 0.72] | 715.9 | 0.64 | .525 | .747 |
|  | Immoral × Empathy | 0.13 | 0.28 | [-0.42, 0.67] | 717.4 | 0.45 | .653 | .963 |
|  | AI × Empathy | 0.25 | 0.38 | [-0.50, 0.99] | 853.1 | 0.65 | .514 | .685 |
|  | Moral × AI × Empathy | -0.56 | 0.40 | [-1.36, 0.23] | 726.4 | -1.39 | .166 | .832 |
|  | Immoral × AI × Empathy | -0.03 | 0.41 | [-0.84, 0.79] | 723.9 | -0.07 | .947 | .968 |
| **Moved** | **Intercept** | **54.54** | **4.58** | **[40.20, 68.88]** | **3.1** | **11.90** | **.001** |  |
|  | Moral | 0.71 | 2.14 | [-3.50, 4.91] | 729.4 | 0.33 | .741 | .746 |
|  | **Immoral** | **-6.44** | **2.15** | **[-10.67, -2.21]** | **729.1** | **-2.99** | **.003** | **.004** |
|  | AI | -3.83 | 3.03 | [-9.79, 2.12] | 783.7 | -1.26 | .206 | .362 |
|  | **Empathy** | **1.71** | **0.41** | **[0.91, 2.52]** | **767.0** | **4.17** | **< .001** | **< .001** |
|  | Moral × AI | -0.18 | 3.05 | [-6.16, 5.80] | 735.2 | -0.06 | .953 | .975 |
|  | Immoral × AI | 2.13 | 3.13 | [-4.02, 8.28] | 736.6 | 0.68 | .497 | .746 |
|  | Moral × Empathy | 0.02 | 0.41 | [-0.79, 0.83] | 727.3 | 0.05 | .957 | .957 |
|  | Immoral × Empathy | -0.35 | 0.41 | [-1.16, 0.46] | 728.3 | -0.84 | .400 | .963 |
|  | AI × Empathy | -1.01 | 0.60 | [-2.19, 0.17] | 789.6 | -1.67 | .095 | .525 |
|  | Moral × AI × Empathy | 0.52 | 0.60 | [-0.66, 1.70] | 736.9 | 0.87 | .385 | .832 |
|  | Immoral × AI × Empathy | 0.89 | 0.62 | [-0.32, 2.09] | 733.9 | 1.44 | .150 | .401 |
| **Beauty** | **Intercept** | **66.72** | **3.18** | **[58.47, 74.97]** | **4.8** | **20.99** | **< .001** |  |
|  | Moral | -2.24 | 2.02 | [-6.20, 1.73] | 731.9 | -1.11 | .268 | .746 |
|  | **Immoral** | **-8.22** | **2.03** | **[-12.21, -4.23]** | **731.4** | **-4.05** | **< .001** | **< .001** |
|  | AI | -5.09 | 2.84 | [-10.67, 0.49] | 791.4 | -1.79 | .073 | .362 |
|  | **Empathy** | **1.65** | **0.39** | **[0.89, 2.40]** | **774.8** | **4.28** | **< .001** | **< .001** |
|  | Moral × AI | 3.09 | 2.87 | [-2.55, 8.72] | 737.4 | 1.07 | .283 | .975 |
|  | Immoral × AI | 3.37 | 2.95 | [-2.43, 9.17] | 738.8 | 1.14 | .254 | .746 |
|  | Moral × Empathy | 0.23 | 0.39 | [-0.54, 0.99] | 729.1 | 0.58 | .560 | .747 |
|  | Immoral × Empathy | -0.02 | 0.39 | [-0.78, 0.75] | 730.1 | -0.05 | .963 | .963 |
|  | AI × Empathy | -0.23 | 0.56 | [-1.33, 0.88] | 797.3 | -0.40 | .688 | .780 |
|  | Moral × AI × Empathy | 0.07 | 0.57 | [-1.04, 1.19] | 738.7 | 0.13 | .895 | .895 |
|  | Immoral × AI × Empathy | -0.29 | 0.58 | [-1.43, 0.85] | 735.7 | -0.50 | .614 | .899 |
| **Connected** | **Intercept** | **55.90** | **2.56** | **[50.49, 61.31]** | **17.1** | **21.80** | **< .001** |  |
|  | Moral | 1.50 | 2.08 | [-2.59, 5.58] | 728.0 | 0.72 | .473 | .746 |
|  | **Immoral** | **-7.17** | **2.10** | **[-11.28, -3.06]** | **728.8** | **-3.42** | **< .001** | **.001** |
|  | AI | -1.72 | 3.10 | [-7.80, 4.37] | 737.2 | -0.55 | .580 | .761 |
|  | **Empathy** | **1.73** | **0.42** | **[0.90, 2.55]** | **721.1** | **4.11** | **< .001** | **< .001** |
|  | Moral × AI | -1.21 | 2.96 | [-7.02, 4.61] | 734.7 | -0.41 | .684 | .975 |
|  | Immoral × AI | 0.62 | 3.05 | [-5.36, 6.61] | 735.5 | 0.20 | .838 | .923 |
|  | Moral × Empathy | 0.34 | 0.40 | [-0.44, 1.13] | 726.5 | 0.85 | .394 | .747 |
|  | Immoral × Empathy | -0.14 | 0.40 | [-0.93, 0.65] | 727.1 | -0.35 | .725 | .963 |
|  | AI × Empathy | -0.52 | 0.61 | [-1.72, 0.69] | 743.8 | -0.84 | .400 | .685 |
|  | Moral × AI × Empathy | 0.15 | 0.58 | [-1.00, 1.30] | 735.2 | 0.26 | .798 | .895 |
|  | Immoral × AI × Empathy | 0.31 | 0.60 | [-0.87, 1.48] | 732.0 | 0.51 | .609 | .899 |
| ***Note.*** CI = confidence interval; df = degrees of freedom calculated using Satterthwaite's method. Adjusted p-value = Benjamini-Hochberg adjusted p-value, corrected across all dependent variables separately. Model 3a includes random intercepts for participants and songs, fixed effects for Morality, Humanness, and Empathy, a two-way interaction between Morality × Humanness, Morality × Empathy, Humanness × Empathy, and a three-way interaction between Morality × Humanness × Empathy. Empathy is mean-centered. Reference categories: Morality = Neutral, Humanness = Human. | | | | | | | | |

**Table S5. Linear Mixed-Effects Model Coefficients Model 3b**

| *Dependent Variable* | *Predictor* | *b* | *SE* | *95% CI* | *df* | *t* | *p*  *(raw)* | *p*  *(adj.)* |
| --- | --- | --- | --- | --- | --- | --- | --- | --- |
| **Creative** | **Intercept** | **63.45** | **1.92** | **[59.68, 67.21]** | **664.6** | **33.10** | **< .001** |  |
|  | Moral | -0.55 | 1.69 | [-3.87, 2.78] | 726.7 | -0.32 | .746 | .746 |
|  | **Immoral** | **-5.91** | **1.71** | **[-9.27, -2.55]** | **726.7** | **-3.46** | **< .001** | **.001** |
|  | AI | -3.57 | 2.73 | [-8.93, 1.80] | 675.9 | -1.31 | .192 | .362 |
|  | **Empathy** | **1.55** | **0.37** | **[0.82, 2.28]** | **663.6** | **4.18** | **< .001** | **< .001** |
|  | Moral × AI | -0.08 | 2.42 | [-4.83, 4.68] | 732.0 | -0.03 | .975 | .975 |
|  | Immoral × AI | -0.24 | 2.50 | [-5.15, 4.66] | 732.9 | -0.10 | .923 | .923 |
|  | Moral × Empathy | 0.28 | 0.33 | [-0.36, 0.93] | 726.1 | 0.86 | .392 | .747 |
|  | Immoral × Empathy | 0.07 | 0.33 | [-0.58, 0.72] | 726.8 | 0.20 | .841 | .963 |
|  | AI × Empathy | -0.41 | 0.54 | [-1.47, 0.66] | 685.6 | -0.75 | .451 | .685 |
|  | Moral × AI × Empathy | 0.27 | 0.48 | [-0.67, 1.21] | 734.4 | 0.56 | .573 | .895 |
|  | Immoral × AI × Empathy | -0.02 | 0.49 | [-0.99, 0.95] | 731.0 | -0.04 | .968 | .968 |
| ***Note.*** CI = confidence interval; df = degrees of freedom calculated using Satterthwaite's method. Adjusted p-value = Benjamini-Hochberg adjusted p-value, corrected across all dependent variables separately. Model 3b includes random intercepts for participants, fixed effects for Morality, Humanness, and Empathy, a two-way interaction between Morality × Humanness, Morality × Empathy, Humanness × Empathy, and a three-way interaction between Morality × Humanness × Empathy. Empathy is mean-centered. Reference categories: Morality = Neutral, Humanness = Human. | | | | | | | | |

**Table S6. Linear Mixed-Effects Model Coefficients Model 4**

| *Dependent Variable* | *Predictor* | *b* | *SE* | *95% CI* | *df* | *t* | *p*  *(raw)* | *p*  *(adj.)* |
| --- | --- | --- | --- | --- | --- | --- | --- | --- |
| **Vitality Index** | **Intercept** | **36.84** | **3.93** | **[27.77, 45.91]** | **7.9** | **9.38** | **< .001** |  |
|  | Moral | -0.75 | 1.84 | [-4.36, 2.87] | 724.3 | -0.41 | .685 | .789 |
|  | **Immoral** | **-4.33** | **1.85** | **[-7.97, -0.69]** | **724.2** | **-2.34** | **.020** | **.023** |
|  | AI | -1.92 | 2.69 | [-7.20, 3.35] | 748.9 | -0.72 | .474 | .542 |
|  | **Age** | **-0.51** | **0.14** | **[-0.80, -0.23]** | **740.9** | **-3.52** | **< .001** | **.004** |
|  | Moral × AI | 0.78 | 2.62 | [-4.36, 5.92] | 729.9 | 0.30 | .766 | .958 |
|  | Immoral × AI | 1.53 | 2.69 | [-3.76, 6.82] | 730.3 | 0.57 | .570 | .778 |
|  | Moral × Age | -0.01 | 0.14 | [-0.28, 0.27] | 723.5 | -0.04 | .967 | .983 |
|  | Immoral × Age | -0.05 | 0.14 | [-0.33, 0.23] | 725.4 | -0.33 | .744 | .959 |
|  | AI × Age | 0.32 | 0.21 | [-0.09, 0.72] | 754.5 | 1.52 | .128 | .713 |
|  | Moral × AI × Age | -0.09 | 0.20 | [-0.48, 0.31] | 729.3 | -0.43 | .670 | .670 |
|  | Immoral × AI × Age | -0.01 | 0.21 | [-0.42, 0.40] | 731.8 | -0.04 | .968 | .968 |
| **Moved** | **Intercept** | **55.36** | **3.41** | **[47.93, 62.79]** | **12.2** | **16.21** | **< .001** |  |
|  | Moral | 0.58 | 2.15 | [-3.64, 4.80] | 728.5 | 0.27 | .789 | .789 |
|  | **Immoral** | **-6.11** | **2.16** | **[-10.36, -1.86]** | **728.1** | **-2.82** | **.005** | **.007** |
|  | AI | -5.23 | 3.08 | [-11.27, 0.81] | 770.5 | -1.70 | .090 | .180 |
|  | **Age** | **-0.42** | **0.17** | **[-0.74, -0.09]** | **762.9** | **-2.52** | **.012** | **.036** |
|  | Moral × AI | -0.57 | 3.06 | [-6.57, 5.44] | 733.8 | -0.19 | .853 | .958 |
|  | Immoral × AI | 1.73 | 3.15 | [-4.45, 7.90] | 734.1 | 0.55 | .583 | .778 |
|  | Moral × Age | 0.00 | 0.16 | [-0.32, 0.33] | 727.3 | 0.02 | .981 | .983 |
|  | Immoral × Age | -0.21 | 0.17 | [-0.54, 0.11] | 729.2 | -1.28 | .202 | .959 |
|  | AI × Age | 0.19 | 0.24 | [-0.28, 0.66] | 776.2 | 0.80 | .426 | .713 |
|  | Moral × AI × Age | -0.25 | 0.24 | [-0.71, 0.22] | 733.0 | -1.04 | .297 | .396 |
|  | Immoral × AI × Age | 0.15 | 0.24 | [-0.32, 0.63] | 735.9 | 0.63 | .529 | .968 |
| **Liking** | **Intercept** | **65.18** | **2.56** | **[57.56, 72.81]** | **3.4** | **25.47** | **< .001** |  |
|  | Moral | -1.63 | 2.11 | [-5.76, 2.51] | 709.1 | -0.77 | .440 | .789 |
|  | **Immoral** | **-8.33** | **2.13** | **[-12.50, -4.15]** | **728.7** | **-3.92** | **< .001** | **< .001** |
|  | AI | -4.76 | 3.05 | [-10.74, 1.23] | 764.5 | -1.56 | .119 | .191 |
|  | **Age** | **-0.35** | **0.16** | **[-0.67, -0.03]** | **757.3** | **-2.14** | **.032** | **.043** |
|  | Moral × AI | 0.79 | 3.01 | [-5.11, 6.69] | 735.3 | 0.26 | .792 | .958 |
|  | Immoral × AI | 2.14 | 3.10 | [-3.94, 8.22] | 739.5 | 0.69 | .490 | .778 |
|  | Moral × Age | 0.14 | 0.16 | [-0.18, 0.46] | 730.3 | 0.87 | .385 | .981 |
|  | Immoral × Age | 0.01 | 0.16 | [-0.31, 0.33] | 733.1 | 0.08 | .936 | .959 |
|  | AI × Age | 0.22 | 0.24 | [-0.24, 0.69] | 770.5 | 0.95 | .341 | .713 |
|  | Moral × AI × Age | -0.35 | 0.23 | [-0.81, 0.10] | 738.7 | -1.53 | .127 | .254 |
|  | Immoral × AI × Age | -0.18 | 0.24 | [-0.65, 0.29] | 740.6 | -0.75 | .453 | .968 |
| ***Note***. CI = confidence interval; df = degrees of freedom calculated using Satterthwaite's method. Adjusted p-value = Benjamini-Hochberg adjusted p-value, corrected across each dependent variable separately. Model 4 was fit to Vitality, Moved, and Liking. It includes random intercepts for participants and songs nested within vocalist sex, fixed effects for Morality, Humanness, and Age, two-way interactions between Morality × Humanness, Morality × Age, Humanness × Age, and a three-way interaction between Morality × Humanness × Age. | | | | | | | | |

**Table S7. Linear Mixed-Effects Model Coefficients Model 4a**

| *Dependent Variable* | *Predictor* | *b* | *SE* | *95% CI* | *df* | *t* | *p*  *(raw)* | *p*  *(adj.)* |
| --- | --- | --- | --- | --- | --- | --- | --- | --- |
| **Sublimity Index** | **Intercept** | **50.00** | **3.59** | **[38.88, 61.11]** | **3.2** | **13.93** | **< .001** |  |
|  | Moral | -0.82 | 1.59 | [-3.93, 2.29] | 727.5 | -0.52 | .605 | .789 |
|  | **Immoral** | **-6.61** | **1.60** | **[-9.74, -3.48]** | **727.5** | **-4.14** | **< .001** | **< .001** |
|  | AI | -4.09 | 2.41 | [-8.81, 0.64] | 719.8 | -1.70 | .090 | .180 |
|  | **Age** | **-0.32** | **0.13** | **[-0.57, -0.06]** | **709.1** | **-2.44** | **.015** | **.036** |
|  | Moral × AI | 1.36 | 2.26 | [-3.07, 5.79] | 733.1 | 0.60 | .548 | .958 |
|  | Immoral × AI | 3.20 | 2.32 | [-1.36, 7.76] | 733.2 | 1.38 | .168 | .778 |
|  | Moral × Age | -0.00 | 0.12 | [-0.24, 0.24] | 726.7 | -0.02 | .983 | .983 |
|  | Immoral × Age | -0.14 | 0.12 | [-0.38, 0.10] | 728.4 | -1.12 | .265 | .959 |
|  | AI × Age | 0.23 | 0.19 | [-0.14, 0.59] | 722.4 | 1.23 | .220 | .713 |
|  | Moral × AI × Age | -0.23 | 0.17 | [-0.57, 0.11] | 733.0 | -1.31 | .189 | .303 |
|  | Immoral × AI × Age | 0.07 | 0.18 | [-0.28, 0.42] | 734.6 | 0.39 | .698 | .968 |
| **Unease Index** | **Intercept** | **19.46** | **3.26** | **[8.59, 30.33]** | **2.8** | **5.96** | **.012** |  |
|  | Moral | 1.64 | 1.45 | [-1.21, 4.49] | 717.3 | 1.13 | .259 | .789 |
|  | **Immoral** | **3.17** | **1.46** | **[0.30, 6.04]** | **718.1** | **2.17** | **.030** | **.030** |
|  | AI | -0.98 | 1.92 | [-4.74, 2.78] | 845.7 | -0.51 | .608 | .608 |
|  | Age | -0.11 | 0.10 | [-0.32, 0.09] | 835.9 | -1.12 | .265 | .265 |
|  | Moral × AI | -0.11 | 2.06 | [-4.16, 3.94] | 724.0 | -0.05 | .958 | .958 |
|  | Immoral × AI | -1.20 | 2.12 | [-5.37, 2.96] | 726.0 | -0.57 | .571 | .778 |
|  | Moral × Age | -0.15 | 0.11 | [-0.37, 0.06] | 716.9 | -1.39 | .165 | .981 |
|  | Immoral × Age | 0.01 | 0.11 | [-0.21, 0.23] | 719.4 | 0.05 | .959 | .959 |
|  | AI × Age | -0.01 | 0.15 | [-0.30, 0.28] | 848.2 | -0.07 | .948 | .948 |
|  | Moral × AI × Age | 0.29 | 0.16 | [-0.02, 0.61] | 724.2 | 1.86 | .063 | .254 |
|  | Immoral × AI × Age | -0.01 | 0.16 | [-0.33, 0.31] | 728.0 | -0.05 | .960 | .968 |
| **Beauty** | **Intercept** | **67.35** | **3.22** | **[59.12, 75.58]** | **5.1** | **20.93** | **< .001** |  |
|  | Moral | -2.27 | 2.03 | [-6.25, 1.71] | 730.7 | -1.12 | .263 | .789 |
|  | **Immoral** | **-8.00** | **2.04** | **[-12.01, -3.99]** | **730.7** | **-3.92** | **< .001** | **< .001** |
|  | AI | -6.49 | 2.93 | [-12.25, -0.74] | 763.6 | -2.21 | .027 | .180 |
|  | **Age** | **-0.37** | **0.16** | **[-0.68, -0.06]** | **753.0** | **-2.36** | **.019** | **.036** |
|  | Moral × AI | 2.58 | 2.89 | [-3.08, 8.25] | 736.4 | 0.90 | .371 | .958 |
|  | Immoral × AI | 3.13 | 2.97 | [-2.70, 8.96] | 736.7 | 1.05 | .292 | .778 |
|  | Moral × Age | 0.11 | 0.15 | [-0.20, 0.41] | 729.4 | 0.69 | .490 | .981 |
|  | Immoral × Age | -0.02 | 0.16 | [-0.33, 0.28] | 731.3 | -0.16 | .874 | .959 |
|  | AI × Age | 0.14 | 0.23 | [-0.30, 0.58] | 766.4 | 0.62 | .535 | .713 |
|  | Moral × AI × Age | -0.40 | 0.22 | [-0.83, 0.04] | 736.0 | -1.80 | .072 | .254 |
|  | Immoral × AI × Age | -0.08 | 0.23 | [-0.53, 0.37] | 738.0 | -0.35 | .723 | .968 |
| ***Note***. CI = confidence interval; df = degrees of freedom calculated using Satterthwaite's method. Adjusted p-value = Benjamini-Hochberg adjusted p-value, corrected across each dependent variable separately. Model 4a was fit to Sublimity, Unease, and Beauty. It includes random intercepts for participants and songs, fixed effects for Morality, Humanness, and Age, two-way interactions between Morality × Humanness, Morality × Age, Humanness × Age, and a three-way interaction between Morality × Humanness × Age. | | | | | | | | |

**Table S8. Linear Mixed-Effects Model Coefficients Model 4b**

| *Dependent Variable* | *Predictor* | *b* | *SE* | *95% CI* | *df* | *t* | *p*  *(raw)* | *p*  *(adj.)* |
| --- | --- | --- | --- | --- | --- | --- | --- | --- |
| **Connected** | **Intercept** | **56.97** | **2.24** | **[52.56, 61.37]** | **701.5** | **25.40** | **< .001** |  |
|  | Moral | 0.72 | 2.08 | [-3.37, 4.81] | 729.0 | 0.35 | .729 | .789 |
|  | **Immoral** | **-7.57** | **2.11** | **[-11.70, -3.43]** | **729.7** | **-3.59** | **< .001** | **< .001** |
|  | AI | -3.55 | 3.20 | [-9.84, 2.74] | 713.8 | -1.11 | .268 | .358 |
|  | Age | -0.29 | 0.17 | [-0.63, 0.05] | 705.0 | -1.68 | .094 | .107 |
|  | Moral × AI | -0.71 | 2.98 | [-6.56, 5.14] | 734.9 | -0.24 | .811 | .958 |
|  | Immoral × AI | 1.05 | 3.08 | [-4.99, 7.08] | 735.8 | 0.34 | .734 | .839 |
|  | Moral × Age | 0.02 | 0.16 | [-0.30, 0.33] | 729.2 | 0.09 | .925 | .983 |
|  | Immoral × Age | -0.12 | 0.16 | [-0.44, 0.20] | 731.0 | -0.73 | .463 | .959 |
|  | AI × Age | 0.05 | 0.25 | [-0.43, 0.54] | 719.5 | 0.22 | .826 | .944 |
|  | Moral × AI × Age | -0.16 | 0.23 | [-0.61, 0.29] | 735.8 | -0.70 | .486 | .555 |
|  | Immoral × AI × Age | 0.07 | 0.24 | [-0.40, 0.54] | 737.6 | 0.29 | .769 | .968 |
| **Creative** | **Intercept** | **64.05** | **1.99** | **[60.15, 67.95]** | **643.6** | **32.25** | **< .001** |  |
|  | Moral | -0.59 | 1.70 | [-3.93, 2.75] | 727.2 | -0.35 | .729 | .789 |
|  | **Immoral** | **-5.87** | **1.72** | **[-9.25, -2.49]** | **727.6** | **-3.41** | **< .001** | **.001** |
|  | AI | -4.85 | 2.83 | [-10.41, 0.72] | 655.1 | -1.71 | .088 | .180 |
|  | **Age** | **-0.35** | **0.15** | **[-0.65, -0.05]** | **646.6** | **-2.28** | **.023** | **.036** |
|  | Moral × AI | -0.45 | 2.44 | [-5.24, 4.33] | 732.4 | -0.19 | .852 | .958 |
|  | Immoral × AI | -0.23 | 2.51 | [-5.17, 4.70] | 732.7 | -0.09 | .926 | .926 |
|  | Moral × Age | 0.10 | 0.13 | [-0.16, 0.36] | 727.2 | 0.77 | .444 | .981 |
|  | Immoral × Age | 0.06 | 0.13 | [-0.20, 0.32] | 728.6 | 0.46 | .647 | .959 |
|  | AI × Age | 0.15 | 0.22 | [-0.28, 0.58] | 660.2 | 0.68 | .495 | .713 |
|  | Moral × AI × Age | -0.29 | 0.19 | [-0.66, 0.08] | 733.2 | -1.56 | .118 | .254 |
|  | Immoral × AI × Age | -0.06 | 0.19 | [-0.45, 0.32] | 734.2 | -0.33 | .742 | .968 |
| ***Note***. CI = confidence interval; df = degrees of freedom calculated using Satterthwaite's method. Adjusted p-value = Benjamini-Hochberg adjusted p-value, corrected across each dependent variable separately. Model 4b was fit to Connected and Creative. It includes random intercepts for participants, fixed effects for Morality, Humanness, and Age, two-way interactions between Morality × Humanness, Morality × Age, Humanness × Age, and a three-way interaction between Morality × Humanness × Age. | | | | | | | | |
